# Asparagine requirement in *Plasmodium berghei* as a target to prevent malaria transmission and liver infections

**DOI:** 10.1038/ncomms9775

**Published:** 2015-11-04

**Authors:** Viswanathan A. Nagaraj, Dhanunjay Mukhi, Vinayagam Sathishkumar, Pradeep A. Subramani, Susanta K. Ghosh, Rajeev R. Pandey, Manjunatha C. Shetty, Govindarajan Padmanaban

**Affiliations:** 1 Department of Biochemistry, Indian Institute of Science, Bangalore 560 012, India; 2 Centre for Infectious Disease Research, Indian Institute of Science, Bangalore 560 012, India; 3 National Institute of Malaria Research (Field Unit), Nirmal Bhawan, ICMR complex, Poojanahalli, Off NH-7, Kannamangala Post, Bangalore 562 110, India

## Abstract

The proteins of *Plasmodium*, the malaria parasite, are strikingly rich in asparagine. *Plasmodium* depends primarily on host haemoglobin degradation for amino acids and has a rudimentary pathway for amino acid biosynthesis, but retains a gene encoding asparagine synthetase (AS). Here we show that deletion of AS in *Plasmodium berghei* (*Pb*) delays the asexual- and liver-stage development with substantial reduction in the formation of ookinetes, oocysts and sporozoites in mosquitoes. In the absence of asparagine synthesis, extracellular asparagine supports suboptimal survival of *Pb*AS knockout (KO) parasites. Depletion of blood asparagine levels by treating *Pb*ASKO-infected mice with asparaginase completely prevents the development of liver stages, exflagellation of male gametocytes and the subsequent formation of sexual stages. *In vivo* supplementation of asparagine in mice restores the exflagellation of *Pb*ASKO parasites. Thus, the parasite life cycle has an absolute requirement for asparagine, which we propose could be targeted to prevent malaria transmission and liver infections.

## Abstract (web summary)

Malaria parasites obtain amino acids primarily from the host, but possess a gene encoding a putative asparagine synthetase. Here, the authors show that this enzyme is functional and that asparagine is crucial for the development of the parasite's sexual stages in mosquitoes and liver stages in mice.

## Graphical abstract

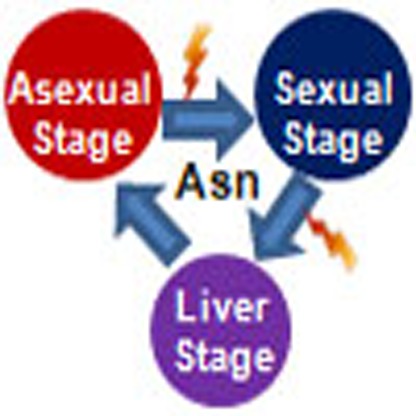

Malaria, transmitted through female *Anopheles* mosquito bites, is a devastating disease that causes close to 0.6 million deaths annually. Of the five *Plasmodium* species that cause human malaria, *Plasmodium falciparum* and *Plasmodium vivax* infections are the most common. While 90% of the malaria-associated deaths occur in Africa due to *P. falciparum* infections, *P. vivax* infections are prevalent in Southeast Asia and South America. With the absence of a highly effective vaccine and with widespread resistance to known antimalarials such as chloroquine and antifolates, artemisinin-based combinations have remained as primary options for malaria therapy^1^,^2^,^3^. However, the emergence of resistance to artemisinin derivatives and various partner drugs such as amodiaquine, mefloquine and piperaquine has been a matter of grave concern^4^,^5^,^6^,^7^. Besides asexual stages for which haemoglobin and hemozoin assimilatory pathways have served as major drug targets^8^,^9^,^10^, malaria eradication would also demand targets to prevent transmission and liver infections^2^. Also, attempts are underway to develop drugs that can offer single-exposure radical cure, prophylaxis and chemoprotection^2^,^11^,^12^. This in turn underscores the need to identify new domains of drug targets that would provide therapeutic options for targeting multiple stages of the parasite cycle.

One such versatile target would be amino acids—molecular building blocks of proteins, precursors of various biologically important molecules and essential sources of carbon, nitrogen and energy metabolism^13^,^14^. *Plasmodium* lacks the canonical pathways for amino acid biosynthesis^15^,^16^ and, therefore, relies mainly on host haemoglobin degradation and extracellular sources to meet its amino acid requirements. Studies carried out using minimal RPMI medium devoid of amino acids have revealed that the blood-stage parasites can survive and proliferate when isoleucine—the only amino acid that is absent in haemoglobin—is provided as a sole exogenous amino acid^17^. This indeed exemplifies that the amino acids derived from haemoglobin degradation are adequate to support the growth when blood-stage parasites are supplemented with isoleucine. Although auxotrophic for most of its amino acids, the parasite genome encodes a few enzymes synthesizing glycine, proline, glutamate, glutamine, aspartate and asparagine, the functional significance of which remains unknown^15^,^16^. Of these amino acids, asparagine plays a pivotal role in the parasite life cycle by serving as one of the most abundant amino acids in *Plasmodium* proteins^18^. While this could be partly explained by the A–T rich nature of *Plasmodium* genomes, the frequency of asparagine is also quite high in the proteins of *P. vivax* and *P. knowlesi* whose genomes are relatively G–C rich^18^,^19^,^20^. Consequently, the malaria parasite has also retained asparagine synthetase (AS) that catalyses the formation of asparagine from aspartate.

AS is an important enzyme as a chemotherapeutic target for acute lymphoblastic leukaemia (ALL)^21^. Since leukaemia cells depend primarily on exogenous asparagine for their proliferation, asparaginase treatment has been successfully carried out in patients with ALL to deplete the malignant cells from asparagine^22^,^23^. Also, there are efforts to develop AS inhibitors that can circumvent the development of resistance towards asparaginase treatment that has been mainly attributed to the upregulation of endogenous AS in leukaemia cells^21^,^24^. Given the proliferating ability of malaria parasites and their analogy to cancer cells together with the abundance of asparagine in the parasite^18^,^25^, it would be interesting to explore the asparagine requirement in malaria parasites as a therapeutic target.

Amino acid requirements in the malaria parasite have been hitherto considered only in the blood stages where haemoglobin serves a major reservoir of amino acids^17^,^26^,^27^,^28^. Here we use *P. berghei* as an *in vivo* rodent parasite model to address the significance of asparagine requirement in the entire life cycle of malaria parasites by performing targeted deletion of endogenous AS and depleting the extracellular asparagine by asparaginase treatment. We show that extracellular asparagine plays a key role in *Pb*AS knockout (*Pb*ASKO)-infected mosquitoes and mice. We propose a combined approach of targeting the endogenous AS and depleting the extracellular asparagine to prevent transmission and liver infections. To the best of our knowledge, this is the first report emphasizing the significance of amino acid requirements and the contribution of extracellular sources in the sexual and liver stages where the parasite development takes place in a haemoglobin-free environment. Our study substantiates the therapeutic potential of amino acid requirements for targeting the multiple stages of malaria parasite life cycle.

## Results

### *P. berghei* AS is enzymatically active

**Figure 1:**
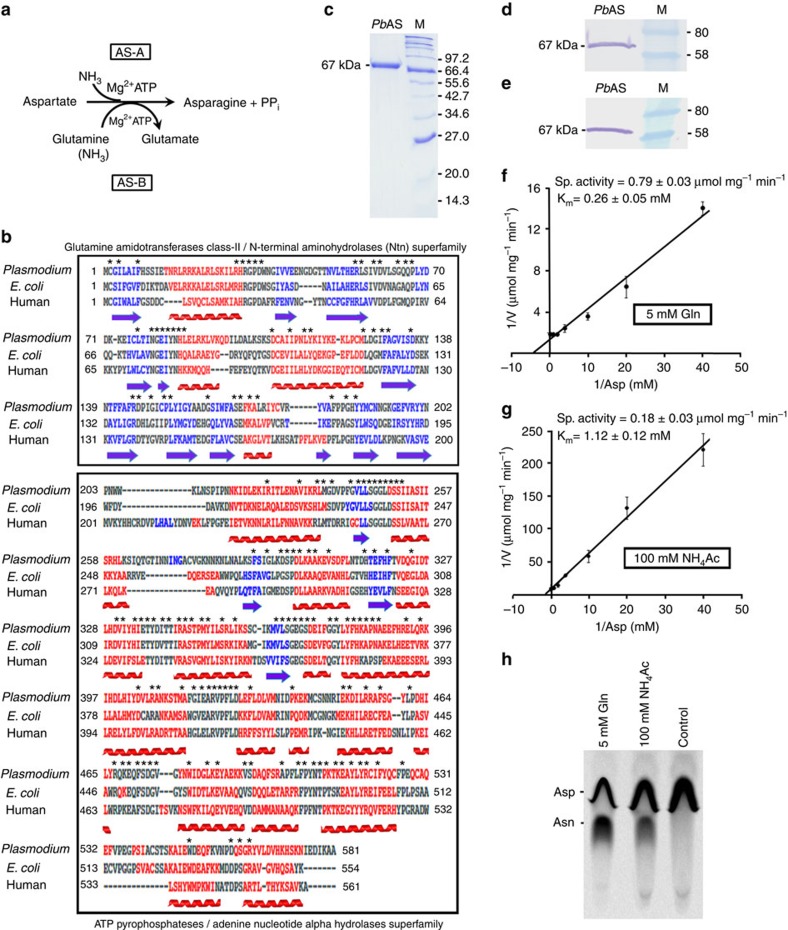
Asparagine synthetase of malaria parasites exhibits AS-B activity. (**a**) Schematic representation of AS-A (ammonia dependent) and AS-B (both glutamine and ammonia dependent) activities. (**b**) Structure-based sequence alignment of *P. berghei* AS (*Plasmodium*) with *E. coli* and human AS-B. α-helices and β-sheets are represented in red and blue, respectively. N-terminal domain (1–202 residues) representing Class-II glutamine amidotransferases/N-terminal aminohydrolases (Ntn) superfamily and C-terminal domain (203–581 residues) representing ATP pyrophosphatases/adenine nucleotide alpha hydrolases superfamily are highlighted in boxes. The residues conserved across all three ASs are marked by asterisks (*). (**c**) SDS–PAGE analysis of purified recombinant *Pb*AS (67 kDa). Lane M: Protein molecular weight marker (kDa). (**d,e**) Western analyses of recombinant *Pb*AS with anti-polyHistidine antibody (**d**) and anti-*Pb*AS IgG (**e**), respectively (full blots are shown in Supplementary Fig. 6). Lane M: Prestained protein molecular weight marker (kDa). (**f,g**) Lineweaver-Burk plots for *Pb*AS with either glutamine (**f**) or ammonia (**g**) as nitrogen source. The assays were carried out using different concentrations of aspartate. (**h**) Radioactive assays performed with [U-^14^C]-aspartate and their product identification by TLC. For control, heat-denatured enzyme was used.

Asparagine biosynthesis is catalysed by two evolutionarily independent families of enzymes—AS-A and AS-B^29^,^30^—of which AS-A catalyses the amidation of aspartate using ammonia as nitrogen source, whereas AS-B can utilize both glutamine and ammonia though glutamine is preferred under physiological conditions (Fig. 1a). Sequence analysis of the annotated *Plasmodium* AS revealed that the parasite enzyme belongs to AS-B, comprising an N-terminal glutamine-hydrolyzing domain representing class-II glutamine amidotransferases/N-terminal aminohydrolases superfamily and a C-terminal domain representing ATP pyrophosphatases/adenine nucleotide alpha hydrolases superfamily^21^. The ammonia released during glutamine hydrolysis traverses an intramolecular tunnel and reaches the active site of C-terminal domain where it reacts with β-aspartyl-AMP intermediate to form asparagine^31^. The critical residues identified based on the crystal structure of *Escherichia coli* AS-B such as Cys1, Arg49, Leu50, Ile52, Val53, Asn74, Gly75, Glu76 and Asp98 (required for positioning and hydrolysis of glutamine), Ser234, Gly235, Gly236, Leu237 and Asp238 (constituting the ATP pyrophosphate loop motif) and Leu232, Ser346, Gly347, Glu352, Tyr357, Lys376, Asp384, Arg387 and Lys449 (interacting with β-aspartyl-AMP intermediate; numbers representing *E. coli* AS-B without N-terminal methionine)^21^,^31^ were found to be well conserved in *Plasmodium* AS (Fig. 1b).

To confirm that the parasite enzyme possesses AS-B activity, *Pb*AS cDNA was cloned into *pET-20b(+)* plasmid (Supplementary Fig. 1a) and the C-terminal His-tagged *Pb*AS was over-expressed in *E. coli* Rosetta2DE3pLysS strain and purified using Ni^2+^-NTA resin (Fig. 1c). Since N-terminal His-tag fusion did not yield an active enzyme, *pET-20b(+)* was chosen to overexpress the recombinant *Pb*AS with C-terminal poly-histidine tag. In western blot analysis, the purified recombinant *Pb*AS (67 kDa) was reactive with both anti-polyHistidine (Sigma-Aldrich) antibody (Fig. 1d) and anti-*Pb*AS IgG (Fig. 1e) purified from polyclonal serum-raised against the recombinant protein. AS-B is known to exist as a homodimer and this seems to be essential for its catalytic activity^21^. To confirm the homogeneity of purified recombinant *Pb*AS and rule out the possibility of *Pb*AS forming a hybrid dimer with *E. coli* AS, matrix-assisted laser desorption/ionization analysis of purified *Pb*AS was carried out in which the tryptic peptides corresponding only to *Pb*AS, but not *E. coli* AS were detected (Supplementary Fig. 1b). Kinetic studies carried out with recombinant *Pb*AS using a coupled assay system^32^ indicated that the parasite enzyme can more efficiently synthesize asparagine when glutamine was provided as an amino group donor. The *K*_m_ value for aspartate and the specific activity of *Pb*AS were found to be 0.26±0.05 mM and 0.79±0.03 μmol mg^−1^ min^−1^, respectively, with glutamine as amino donor (Fig. 1f). The corresponding values for aspartate with ammonia as amino donor were 1.12±0.12 mM and 0.18±0.03 μmol mg^−1^ min^−1^, respectively (Fig.1g). The catalytic efficiency of *Pb*AS for aspartate in the presence of glutamine (*k*_cat_/*K*_m_=2.7 × 10^3^ M^−1^ s^−1^) was found to be similar to that of human AS for which the reported *K*_m_ and *k*_cat_/*K*_m_ values were 0.38±0.03 mM and 3.4 × 10^3^ M^−1^ s^−1^, respectively^32^. Given the constraints associated with coupled assay system in crude lysates, a simple radioactive assay with [U-^14^C]-aspartate was developed and the formation of radiolabelled asparagine was detected by thin-layer chromatography (TLC) as well (Fig. 1h). Similar results were also obtained with recombinant *Pf*AS, which shares 77% identity and 86% similarity with *Pb*AS (Supplementary Fig. 2). All these data clearly demonstrated that the AS of malaria parasite is enzymatically active.

### Targeted deletion disrupts the chromosomal locus of *Pb*AS

**Figure 2:**
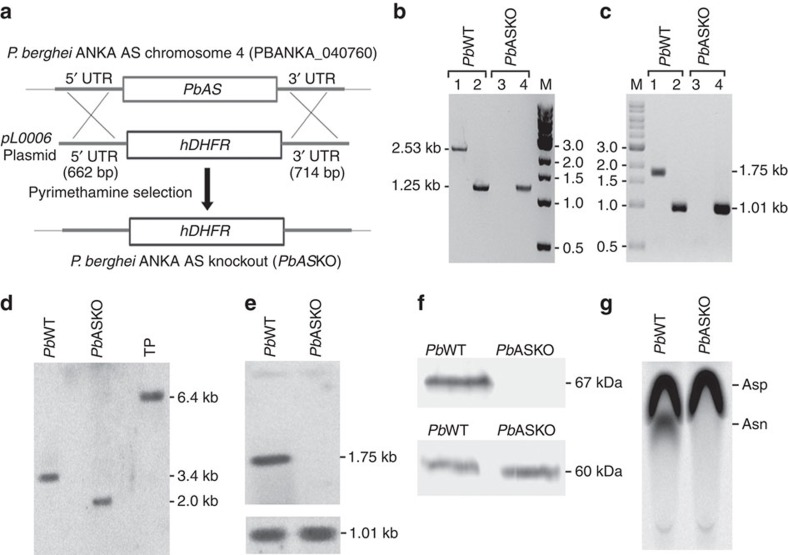
Generation of AS knockout in *P. berghei*. (**a**) Double cross-over recombination strategy followed to generate *Pb*ASKO. (**b**) PCR analysis for genomic DNA isolated from *Pb*WT and *Pb*ASKO parasites. Lane 1 and 3: PCR with *Pb*AS-specific primers; Lane 2 and 4: PCR with *Pb*GAPDH-specific primers (control); Lane M: 1 kb DNA ladder. (**c**) RT-PCR analysis for RNA isolated from *Pb*WT and *Pb*ASKO parasites. Lane 1 and 3: RT-PCR with *Pb*AS-specific primers; Lane 2 and 4: RT-PCR with *Pb*GAPDH specific primers (control); Lane M: 1 kb DNA ladder. (**d**) Southern analysis for genomic DNA isolated from *Pb*WT and *Pb*ASKO parasites. Transgenic plasmid (TP) was also included as control to rule out the presence of any episomes. Genomic DNA preparations and TP were digested with *BsrGI* and *XbaI* followed by hybridization with 5′-UTR specific probe. (**e**) Northern analysis for RNA isolated from *Pb*WT and *Pb*ASKO parasites indicating the absence of AS mRNA (1.75 kb) in *Pb*ASKO. For control, GAPDH (1.01 kb; lower panel) was used (full blots are shown in Supplementary Fig. 6). (**f**) Western blot analysis indicating the absence of AS (67 kDa) in *Pb*ASKO parasite lysate (Supplementary Fig. 6). Parasite lysates of *Pb*WT and *Pb*ASKO containing 100 μg of total protein were used. For control, hsp60 (60 kDa) was used (lower panel). (**g**) Enzyme assays for *Pb*WT and *Pb*ASKO parasites using [U-^14^C]-aspartate. 200 μg of total protein was used per assay.

To determine the significance of AS in the parasite life cycle, chromosomal locus of AS was targeted through double cross-over recombination using *pL0006* plasmid in which 5′- and 3′-untranslated regions (UTRs) of AS were cloned on either side flanking human DHFR selection cassette (Fig. 2a). Ten days post transfection, limiting dilution was carried out for pyrimethamine-resistant parasites to select the individual clones^33^. PCR and reverse transcriptase–PCR (RT-PCR) analyses performed with genomic DNA and RNA isolated from *Pb* wild-type (WT) and *Pb*ASKO parasites revealed the absence of 2.53 and 1.75 kb products in *Pb*ASKO suggesting the targeted deletion of *Pb*AS locus (Fig. 2b,c). This was confirmed by Southern analysis wherein respective products of 3.4 and 2.0 kb were detected for *Pb*WT and *Pb*ASKO parasites (Fig. 2d). Also, northern (Fig. 2e) and western (Fig. 2f) analyses were carried out to ensure the absence of AS mRNA (1.75 kb) and AS protein (67 kDa) in *Pb*ASKO. This was further substantiated with enzyme assays using [U-^14^C]-aspartate where the formation of asparagine could be detected in *Pb*WT, but not in *Pb*ASKO parasites (Fig. 2g). Thus, the chromosomal locus of AS in *P. berghei* was successfully targeted to generate the KO parasites.

### AS is essential for optimal *in vivo* growth of *P. berghei*

**Figure 3:**
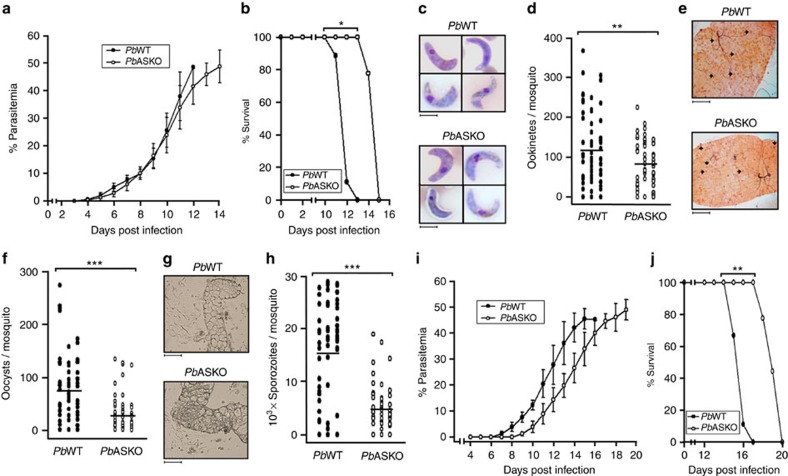
*In vivo* growth phenotype analyses for asexual, sexual and liver stages of *Pb*ASKO. (**a**) Growth analyses for intra-erythrocytic development of *Pb*WT and *Pb*ASKO parasites in mice. The percentage of parasitemia was calculated as described in Methods. The data represent Mean±s.d. values obtained for nine mice from three different batches. (**b**) Percentage survival of mice infected with intra-erythrocytic stages of *Pb*WT and *Pb*ASKO parasites. The cumulative percentage survival was calculated from nine mice that were used for intra-erythrocytic growth analyses. (**c**) Ookinete images of *Pb*WT and *Pb*ASKO parasites stained with Giemsa reagent. Scale bar, 5 μm (**d**) Quantification of *Pb*WT and *Pb*ASKO ookinetes formed *in vivo*. The average number of ookinetes obtained for *Pb*WT and *Pb*ASKO were 119 and 79, respectively. (**e**) *Pb*WT and *Pb*ASKO oocysts stained with mercurochrome. Five mature oocysts from *Pb*WT- and *Pb*ASKO-infected midguts are indicated by arrows. Scale bar, 50 μm. (**f**) Quantification of *Pb*WT and *Pb*ASKO oocysts. The average numbers of *Pb*WT and *Pb*ASKO oocysts were 76 and 30, respectively. (**g**) Bright field images for salivary glands dissected from *Pb*WT- and *Pb*ASKO-infected mosquitoes. Scale bar, 100 μm. Thread-like structures surrounding the lobes of salivary glands represent sporozoites. (**h**) Quantification of *Pb*WT and *Pb*ASKO sporozoites. The average number of sporozoites detected in *Pb*WT and *Pb*ASKO were 15,351 and 4,825, respectively. The data provided for ookinete, oocyst and sporozoite quantifications represent the values obtained for 60 mosquitoes from three different batches. (**i**) Liver-stage development of *Pb*WT and *Pb*ASKO sporozoites. The data represent the Mean±s.d. values of nine mice from three different batches. (**j**) Cumulative percentage survival of mice infected with *Pb*WT and *Pb*ASKO sporozoites. *P<0.05, **P<0.01 and ***P<0.001 (two-tailed unpaired Student's *t*-test).

To assess the phenotype of *Pb*ASKO parasites, growth curve analyses for intra-erythrocytic development (asexual stages) were carried out by injecting mice intraperitoneally with 10^5^ *Pb*WT or *Pb*ASKO asexual-stage parasites. The data presented in Fig. 3a,b indicated a significant growth difference between *Pb*WT and *Pb*ASKO parasites with 77% of *Pb*ASKO-infected mice surviving on day 14 in comparison with 88% of *Pb*WT-infected mice that survived only till day 11. The results also revealed that the *Pb*ASKO-infected mice could sustain higher parasitemia for at least 3 more days. For sexual-stage analyses, *Anopheles stephensi* mosquitoes were allowed to feed on mice infected with *Pb*WT or *Pb*ASKO parasites. Although *Pb*ASKO parasites could form ookinetes (Fig. 3c), oocysts (Fig. 3e) and sporozoites (Fig. 3g), there were significant reductions in their numbers with respect to *Pb*WT. While the formation of ookinetes was reduced by 33% (Fig. 3d), the respective reductions for oocysts and sporozoites were found to be 61 and 69% (Fig. 3f,h). However, no significant abnormalities associated with male (micro-) and female (macro-) gametocytes of *Pb*ASKO could be observed in terms of their morphology, numbers and exflagellation (Supplementary Fig. 3; Supplementary Movies 1 and 2). To independently analyse the liver-stage development, naive Swiss mice were injected intravenously with 10^4^ *Pb*WT or *Pb*ASKO sporozoites and the subsequent appearance of asexual-stage parasites in blood was examined. The data presented in Fig. 3i,j revealed a significant delay in the liver-stage development of *Pb*ASKO sporozoites for which the asexual stages were detectable in the peripheral blood on day 8. This was in contrast to the emergence of *Pb*WT asexual stages that were detectable within 6 days. This delay was further reflected in the mortality of *Pb*ASKO-infected mice of which 45% of them could survive till day 19, almost 3 days longer than that of *Pb*WT. All these results suggested that the parasite's genome-encoded AS is essential for the optimal *in vivo* growth and competency of *P. berghei* to complete its life cycle.

### Asparaginase blocks *Pb*ASKO sexual- and liver-stage formation

**Figure 4:**
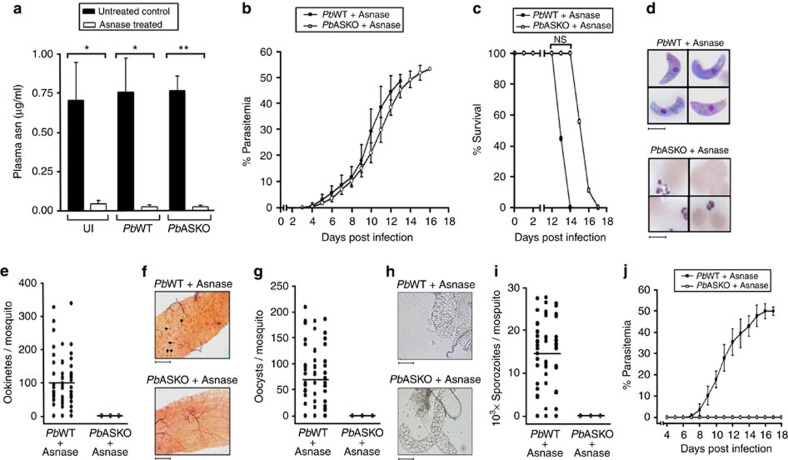
Effect of asparaginase treatment on *in vivo* growth of *Pb*ASKO. (**a**) Quantification of plasma asparagine levels in *Pb*WT-infected and *Pb*ASKO-infected mice treated with asparaginase (Asnase). UI, uninfected mice. (**b**) Growth curves of *Pb*WT and *Pb*ASKO asexual-stage parasites in mice treated with asparaginase. Mice were injected intraperitoneally with 10^5^ parasites and the percentage of parasitemia was calculated. The data represent mean±s.d. values obtained for nine mice from three different batches. (**c**) Percentage survival of mice infected with *Pb*WT and *Pb*ASKO asexual-stage parasites and treated with asparaginase. The cumulative percentage survival was calculated from nine mice used for growth analyses. (**d**) Examination of ookinete formation in mosquitoes fed on mice infected with *Pb*WT and *Pb*ASKO parasites treated with asparaginase. Scale bar, 5 μm. (**e**) Quantification of ookinetes. (**f**) Examination of oocysts formation. Five mature oocysts from asparaginase-treated *Pb*WT-infected midguts are indicated by arrows. Scale bar, 50 μm. (**g**) Quantification of oocysts. (**h**) Bright field images for salivary glands of asparaginase-treated *Pb*WT- and *Pb*ASKO-infected mosquitoes. Scale bar, 100 μm. (**i**) Quantification of sporozoites. An average of 101 ookinetes, 71 oocysts and 14,000 sporozoites were observed for *Pb*WT parasites treated with asparaginase. *Pb*ASKO treated with asparaginase did not undergo sexual-stage development. The data provided for ookinete, oocyst and sporozoite quantifications represent the values obtained for 60 mosquitoes from three different batches. (**j**) Liver-stage development of *Pb*WT and *Pb*ASKO sporozoites in mice treated with asparaginase. Mice were injected intravenously with 10^4^ sporozoites and the appearance of asexual stages was monitored. The data represent mean±s.d. values of nine mice from three different batches. *P<0.05, **P<0.01 and NS, not significant (two-tailed unpaired Student's *t*-test).

To examine whether extracellular asparagine is critical for the survival of *Pb*ASKO parasites lacking endogenous asparagine synthesis, mice infected intraperitoneally with 10^5^ *Pb*WT or *Pb*ASKO asexual-stage parasites were treated with three doses of L-asparaginase (50 IU per mouse) on day 5, 6 and 7. Liquid chormatography-selected reaction monitoring/mass spectrometry–based quantification of plasma asparagine (Supplementary Fig. 4) on day 8 revealed more than 90% reduction in the plasma asparagine levels of mice treated with asparaginase (Fig. 4a). However, the growth curves obtained for *Pb*WT and *Pb*ASKO (Fig. 4b) intra-erythrocytic development indicated that there was only a slight and statistically insignificant (two-tailed unpaired Student's *t*-test) delay of 1–2 days in overall survival of mice after asparaginase treatment (Fig. 4c). For sexual-stage development analyses, mosquito-feeding experiments were carried out on day 8 using asparaginase-treated mice. While asparaginase treatment *per se* did not affect the formation of sexual stages in *Pb*WT-infected mosquitoes, no ookinetes, oocysts or sporozoites could be detected in mosquitoes fed on *Pb*ASKO-infected mice treated with asparaginase (Figs 4d–i). Even more interestingly, asparaginase treatment completely prevented the liver-stage development of *Pb*ASKO sporozoites when 10^4^ sporozoites were injected on day 1 into mice treated with three doses of asparaginase on day 0, 1 and 2. However, asparaginase treatment did not prevent the liver-stage development of *Pb*WT sporozoites and the asexual-stage parasites could be readily detected in blood from day 6 onwards (Fig. 4j). The data obtained suggested that the extracellular asparagine is essential for the sexual and liver-stage development of *Pb*ASKO parasites.

### Asparaginase prevents *Pb*ASKO male gametocyte exflagellation

**Figure 5:**
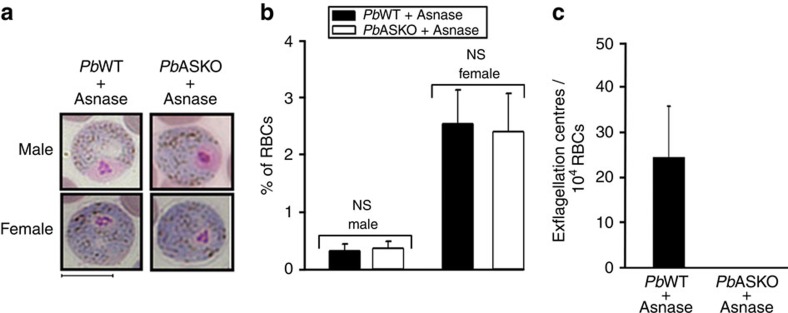
Assessment of gametocytes in *Pb*WT-infected and *Pb*ASKO-infected mice treated with asparaginase. (**a**) Bright field images for male (micro-) and female (macro-) gametocytes of *Pb*WT and *Pb*ASKO stained with Giemsa reagent. Mice were treated with three doses of asparaginase (Asnase) and blood smears were prepared on day 8 post infection. Scale bar, 5 μm. (**b**) Quantification of gametocytes. Based on morphology, Giemsa-stained male and female gametocytes were identified and counted. NS, not significant (two-tailed unpaired Student's *t*-test). (**c**) Exflagellation centres observed in blood collected from *Pb*WT- and *Pb*ASKO-infected mice treated with asparaginase. The mean±s.d. values were obtained from nine mice.

The absence of ookinete formation in mosquitoes fed with *Pb*ASKO parasites from mice treated with asparaginase indicated the possibility of defective gametocytes. To address this, the morphology and numbers of *Pb*ASKO male and female gametocytes formed in mice treated with three doses of asparaginase were analysed. The results presented in Fig. 5a,b suggested that the asparaginase treatment had no detectable effect on the morphology of *Pb*ASKO gametocytes and their numbers were comparable to the gametocytes formed in *Pb*WT-infected mice treated with asparaginase. Interestingly, *Pb*ASKO male gametocytes from asparaginase-treated mice failed to undergo exflagellation, when the blood collected from such mice were examined *in vitro* (Fig. 5c; Supplementary Movie 3). This was in contrast to the asparaginase-treated *Pb*WT male gametocytes that were able to exflagellate (Fig. 5c; Supplementary Movie 4). These data suggested that the extracellular asparagine is vital for *Pb*ASKO male gametocyte exflagellation.

### Asparaginase prevents *Pb*ASKO EEF formation

**Figure 6:**
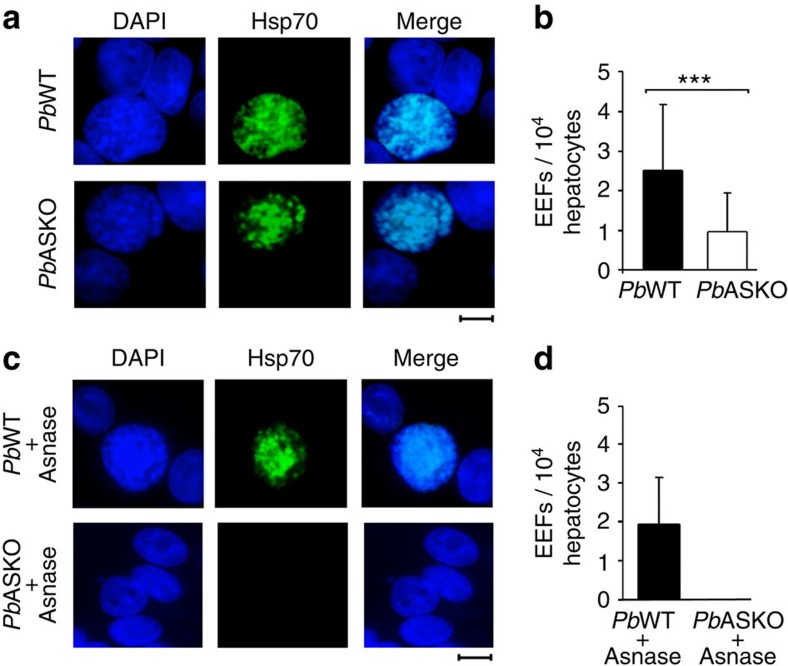
*In vivo* development of *Pb*ASKO EEFs in mice treated with and without asparaginase. (**a**) Immunofluorescence images of *Pb*WT and *Pb*ASKO EEFs. DAPI, FITC (*Plasmodium* hsp70) and merge images are presented for hepatocytes isolated from mice infected with *Pb*WT and *Pb*ASKO sporozoites. Scale bar, 10 μm. (**b**) Quantification of EEFs. Hepatocytes isolated from three different mice infected with 10^5^ *Pb*WT or *Pb*ASKO sporozoites were pooled. The data represent Mean±s.d. values of 30 independent readings. (**c**) Immunofluorescence images of *Pb*WT and *Pb*ASKO EEFs treated with asparaginase (Asnase). DAPI, FITC (*Plasmodium* hsp70) and merge images are presented for hepatocytes isolated from asparaginase-treated mice infected with *Pb*WT and *Pb*ASKO sporozoites. Scale bar, 10 μm. (**d**) Quantification of EEFs. Hepatocytes isolated from three different mice infected with 10^5^ *Pb*WT or *Pb*ASKO sporozoites and treated with asparaginase were pooled. The data represent mean±s.d. values of 30 independent readings. ***P<0.001 (two-tailed unpaired Student's *t*-test).

To analyse the liver-stage development of *Pb*ASKO sporozoites with and without asparaginase treatment, immunofluorescence analysis of hepatocytes isolated from mice infected with 10^5^ *Pb*ASKO sporozoites using *Plasmodium*-specific hsp70 antibodies was carried out. For control, hepatocytes isolated from mice infected with 10^5^ *Pb*WT sporozoites were used. These studies revealed the existence of exo-erythrocytic forms (EEFs) after 50 h post-infection with *Pb*WT- and *Pb*ASKO-infected hepatocytes (Fig. 6a). However, the number of EEFs quantified from *Pb*ASKO-infected hepatocytes was found to be significantly less when compared with *Pb*WT-infected hepatocytes (Fig. 6b). This in turn suggested that the delay observed in the appearance of *Pb*ASKO asexual stages on injection of sporozoites in mice (Fig. 3i) was due to the decreased formation of *Pb*ASKO EEFs in the liver. Interestingly, EEFs for *Pb*ASKO sporozoites were not detected in hepatocytes isolated from *Pb*ASKO sporozoite-infected mice treated with asparaginase (Fig. 6c,d) and this was in agreement with the absence of asexual stages in *Pb*ASKO sporozoite-infected mice treated with asparaginase (Fig. 4j). The absence of liver stage development in *Pb*ASKO sporozoite-infected mice treated with asparaginase was also confirmed by performing RT-PCR analysis for total RNA isolated from infected mouse liver samples wherein the product corresponding to parasite GAPDH could not be detected (Supplementary Fig. 5). The results obtained revealed that extracellular asparagine is essential for the *in vivo* formation of *Pb*ASKO EEFs.

### *In vivo* asparagine injection restores *Pb*ASKO exflagellation

**Figure 7:**
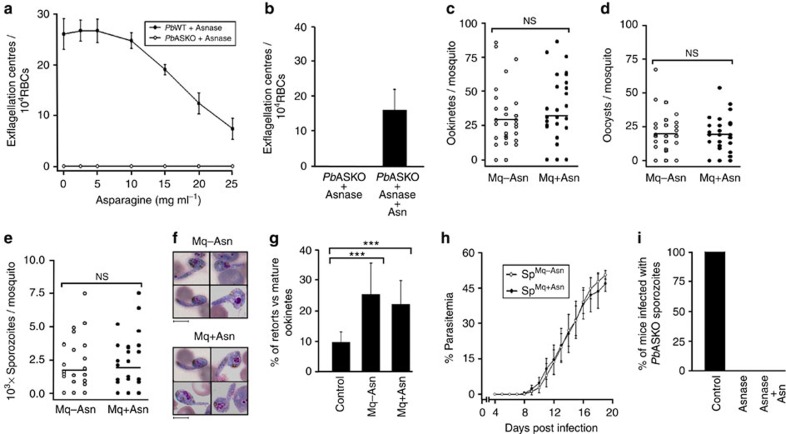
Effect of asparagine supplementation in *Pb*ASKO parasites treated with aspraginase. (**a**) Exflagellation of asparaginase-treated *Pb*WT and *Pb*ASKO male gametocytes in medium containing different concentrations of asparagine. Blood samples collected from three different mice treated with asparaginase were used. Mean±s.d. values were calculated for the respective concentrations. (**b**) Exflagellation centres observed in asparaginase-treated *Pb*ASKO-infected mice with and without the supplementation of asparagine. The data represent the values obtained from nine mice from three different batches. (**c**–**e**) Quantification of ookinetes, oocysts and sporozoites in mosquitoes fed on exflagellation restored *Pb*ASKO-infected mice treated with asparaginase. Mq−Asn, mosquitoes without asparagine supplementation (control); Mq+Asn, mosquitoes supplemented with asparagine. The data represent 30 mosquitoes from three different batches. The average values obtained for ookinetes, oocysts and sporozoites were 31, 19 and 1,794 for Mq−Asn and 34, 18 and 2,122 for Mq+Asn, respectively. NS, not significant. (**f**) Giemsa-stained images of retorts observed in Mq−Asn and Mq+Asn. (**g**) Quantification of retorts. For control, mosquitoes fed on mice infected with *Pb*ASKO parasites were used. ***P values (two-tailed unpaired Student's *t*-test) for Mq−Asn and Mq+Asn with respect to the control were <0.001. (**h**) Liver-stage development in mice infected with Mq-Asn and Mq+Asn sporozoites. 10^4^ sporozoites were injected intravenously. Sp^Mq−Asn^, sporozoites collected from Mq-Asn; Sp^Mq+Asn^, sporozoites collected from Mq+Asn; (**i**) Effect of asparagine supplementation on liver-stage development of *Pb*ASKO sporozoites treated with asparaginase. For control, mice without asparaginase treatment were used. The data represent nine mice from three different batches. Asnase, asparaginase.

Studies were also carried out with asparaginase-treated *Pb*ASKO parasites to assess the effects of *in vitro* and *in vivo* asparagine supplementation. To determine whether extracellular asparagine was only required for exflagellation as such or indeed essential for the process of maturation of *Pb*ASKO male gametocytes, *in vitro* exflagellation of *Pb*ASKO male gametocytes present in the blood collected from mice treated with asparaginase was examined with different concentrations of asparagine added to the exflagellation medium. For control, *Pb*WT gametocytes treated with asparaginase were used. While a concentration up to a maximum of 5 mg ml^−1^ did not affect the exflagellation of asparaginase-treated *Pb*WT gametocytes, inhibition was observed at higher concentrations. However, none of the concentrations used were able to restore the exflagellation of *Pb*ASKO male gametocytes obtained from mice treated with asparaginase (Fig. 7a). These results suggested that asparagine is essential for the entire process of male gametocyte maturation and its requirement is not just confined to exflagellation.

Since exflagellation of asparaginase-treated *Pb*ASKO male gametocytes could not be restored *in vitro*, asparagine supplementation was attempted *in vivo* to neutralize the effect of asparaginase by injecting asparaginase-treated mice with six respective doses of 2, 4 and 6 mg asparagine through intraperitoneal route for every 12 h starting from the first dose of asparaginase. Given the limitations associated with the solubility of asparagine and the volume that can be injected intraperitoneally, supplementing mice with concentrations higher than 6 mg per mouse did not seem to be realistic. Although none of the concentrations mentioned above could restore the exflagellation with three doses of asparaginase, there was restoration with 6 mg of asparagine when asparaginase treatment was reduced to two doses administered on day 5 and 6 (Fig. 7b; Supplementary Movie 5). The restoration was found to be around 59% in comparison with *Pb*ASKO parasites, which did not receive asparaginase treatment (Supplementary Fig. 3c). Moreover, the reduction of asparaginase treatment was made because of the high activity of asparaginase used in ALL therapy and even two doses were found to be sufficient for inhibiting *Pb*ASKO exflagellation.

To examine whether asparagine supplementation is also required for sexual-stage development in mosquitoes, mosquitoes fed on asparaginase-treated mice in which the *Pb*ASKO exflagellation was restored by asparagine were subjected to asparagine supplementation (0.5% w/v) in the feeding solution. While partial restoration observed in *Pb*ASKO exflagellation was reflected in the formation of ookinetes, oocysts and sporozoites, no significant differences could be observed with respect to the control mosquitoes that did not receive asparagine supplementation (Fig. 7c–e). The decrease in the formation of ookinetes was found to be accompanied by an increase in the percentage of retorts as well (Fig. 7f,g). Further, *Pb*ASKO sporozoites obtained with and without the supplementation of asparagine in mosquitoes showed a similar pattern of liver-stage development in mice (Fig. 7h) with a delay in the appearance of asexual stages. All these results suggested that the restoration of exflagellation was adequate for asparaginase-treated *Pb*ASKO parasites to complete its life cycle. Interestingly, *in vivo* asparagine supplementation capable of restoring the exflagellation of *Pb*ASKO male gametocytes did not support the liver-stage development (Fig. 7i) when mice infected with 10^4^ *Pb*ASKO sporozoites were treated with two doses of asparaginase on day 0 and day 1 followed by supplementation with six doses of asparagine (6 mg per mouse; 12 h interval). This in turn suggested that the availability of supplemented asparagine to the exo-erythrocytic stages whose development occurs within the milieu of metabolically active hepatocytes might differ from the gametocyte development in blood stages. While two doses of asparaginase were equally effective in inhibiting the exflagellation (Fig. 7b) and liver-stage development (Fig. 7i) of *Pb*ASKO parasites, single dose was found to be insufficient.

## Discussion

We show here that asparagine is critical for malaria parasite survival, in line with the known remarkable abundance of asparagine in *Plasmodium* proteins. While the presence of asparagine repeats in low complexity regions exists as a hallmark of *P. falciparum* proteins^34^,^35^, asparagine serves as one of the most abundant amino acids in proteins of other *Plasmodium* species as well^18^. In comparison with prokaryotic and eukaryotic proteomes with an asparagine frequency of ∼4–5% (ref. 36), the frequency of asparagine calculated from the complete coding sequences of *Plasmodium* species was found to fall within a range of 7–14% (*P. falciparum* ∼14%, *P. knowlesi* ∼8%, *P. vivax* ∼7*%, P. berghei* ∼13%, *P. yoelii* ∼13% and *P. chaubadi* ∼12%) with *P. falciparum* presenting the highest degree of asparagine content^18^. While the functional significance of asparagine repeats in *P. falciparum* still remains unclear^37^,^38^, it has been proposed that such asparagine repeats may serve as transfer RNA (tRNA) sponges that can facilitate protein folding by serving as inherent chaperones and control stage-specific expressions by modulating the translational rate^19^. Also, the parasite genome encodes a putative AS despite lacking canonical pathways for amino acid biosynthesis^15^,^16^. In the present study, we have characterized the AS of malaria parasites, generated KO parasites in *P. berghei* to address the essentiality of AS in the entire life cycle and determined the importance of extracellular asparagine in the survival of *Pb*ASKO parasites by using asparaginase treatment to deplete asparagine.

While ammonia-dependent AS-A is known to be present in prokaryotes^29^,^39^,^40^ and in certain protozoan parasites such as *Leishmania* and *Trypanosoma*^41^, AS-B which utilizes glutamine as its preferred amino group donor is found in *E. coli*^31^, yeasts^42^ and higher eukaryotes^32^,^43^. Here we use sequence comparison together with the enzyme assays performed for recombinant *Pb*AS and total parasite lysates to reveal that the parasite enzyme belongs to the AS-B family^30^,^31^,^32^. The catalytic efficiency of the recombinant parasite enzyme with respect to glutamine (*k*_cat_/*K*_m_=2.7 × 10^3^ M^−1^ s^−1^) was found to be at least 14-fold higher in comparison with ammonia (*k*_cat_/*K*_m_=0.19 × 10^3^ M^−1^ s^−1^). Gene KO carried out for AS in *P. berghei* and subsequent comparisons made between the *Pb*ASKO phenotype and *Pb*WT for the entire life cycle showed a significant delay in the mortality of mice infected with *Pb*ASKO asexual-stage parasites. These results serve as evidence of the synthesis of asparagine, suggesting that asparagine derived from haemoglobin degradation and extracellular sources^17^,^26^ may not support optimal blood-stage development. More importantly, the sexual stage development of *Pb*ASKO parasites was drastically affected, with a nearly 69% reduction in the formation of sporozoites observed. We also found a significant decline in the formation of *Pb*ASKO EEFs, which led to a delay in the appearance of asexual stages and subsequent mortality when *Pb*ASKO sporozoites were injected intravenously to initiate liver-stage development in mice. Thus, asparagine synthesis mediated by endogenous AS is crucial for the optimal virulence of malaria parasites especially in the sexual and liver stages where asparagine availabilities in the mosquito host and mouse hepatocytes may prove severely limiting.

Unlike asexual stages wherein haemoglobin degradation serves as an intrinsic source of amino acids^17^,^26^, free-living sexual-stage parasites depend primarily on the mosquito haemolymph, and liver-stage parasites must subvert the metabolically active host hepatocytes for extracellular sources^44^. Given this context, we were interested to determine whether the absence of endogenous asparagine synthesis is compensated through the utilization of extracellular asparagine and this is in turn responsible for the suboptimal survival of *Pb*ASKO sexual and liver stages. To deplete the extracellular asparagine, *Pb*ASKO-infected mice were injected with three doses of L-asparaginase (50 IU per mouse), conforming to the ALL treatment regimens in children and adults that involve multiple doses of L-asparaginase (*E. coli*, *Erwinia* or pegylated)^22^,^23^ administered over several days (5,000–10,000 IU m^−2^ for *E. coli* asparaginase)^22^,^23^,^45^. Although asparaginase treatment did not have significant effect on *Pb*ASKO asexual-stage development *per se*, sexual-stage development was fully inhibited in mosquitoes, as the *Pb*ASKO male gametocytes failed to exflagellate.

While asparagine depletion can affect protein synthesis in general, the expression of a particular subset of proteins related to exflagellation may be hindered severely. In addition, given limitations associated with female gametocyte functionality assessments, abnormalities associated with female gametocytes cannot be ruled out at this stage, and this would require extensive cross-fertilization studies^46^. Interestingly, asparagine content seems to be higher in gametocyte and sporozoite proteins of *P. falciparum* when compared with the asexual stages^19^. Unlike the asexual stages, which are completed within 24 h (for *P. berghei*) followed by the release of merozoites that invade fresh RBCs/reticulocytes, gametocytes reside within the same host cell for several days until gametes form in the mosquito host^47^,^48^. Therefore, their amino acid requirements may differ from those of asexual stages, and especially when the host haemoglobin reservoir is depleted during later stages of maturation. Asparagine depletion was found to inhibit the liver-stage development of *Pb*ASKO sporozoites in mice, suggesting the key role played by asparagine during the liver stages as well. The absence of EEFs and detectable parasite RNA in hepatocytes isolated from mice infected with *Pb*ASKO sporozoites suggests that asparagine depletion may lead to the prevention of *Pb*ASKO sporozoite invasion or to an early arrest of liver-stage development. Further investigations are needed to decipher the molecular events underlying the inhibition of sexual- and liver-stage development as well as the significance of these findings with other *Plasmodium* species, *P. vivax* in particular.

Our next goal was to examine whether asparagine supplementation can rescue effects of asparaginase treatment on *Pb*ASKO parasites. While *in vitro* supplementation had no effect, exflagellation could be partially restored by reducing asparaginase treatment in mice to 2 days and by supplementing them *in vivo* with asparagine, suggesting that asparagine is vital for the functional maturation of male gametocytes rather being required transiently for exflagellation. However, a similar *in vivo* supplementation failed to restore the liver-stage development of *Pb*ASKO sporozoites in asparaginase-treated mice. Considering the development of liver stages in metabolically active hepatocytes and the robust activity of asparaginase utilized in ALL treatment^22^,^23^,^45^, supplemented asparagine may become insufficient and/or less accessible for liver stages. This also denotes that asparagine requirement can serve as an effective target for preventing liver infections.

**Figure 8:**
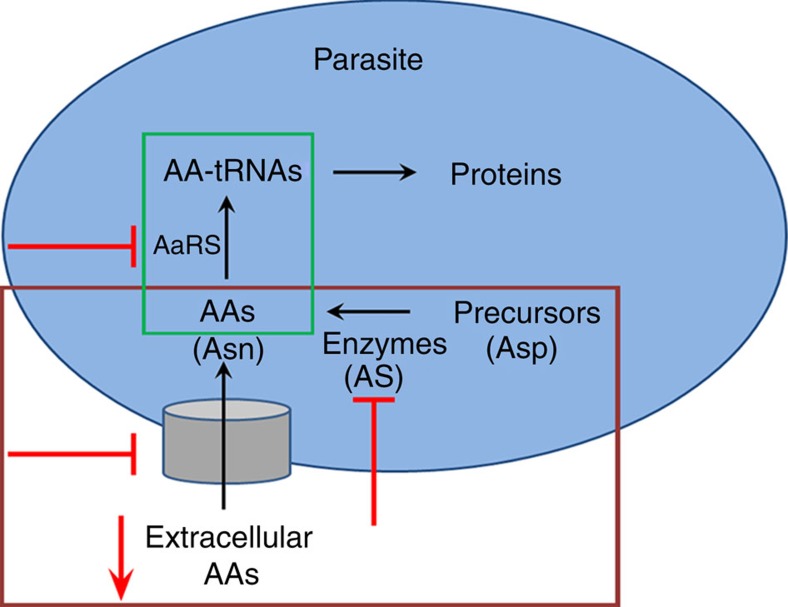
Targeting amino acid requirements in malaria parasites. Therapeutic options of depleting extracellular sources, targeting biosynthetic enzymes, transporters and AaRS are depicted in red. Brown box highlights the approaches relevant for sexual and liver stages. The approach of targeting AaRS is highlighted in green box. AAs, amino acids; AaRS, aminoacyl-tRNA synthetases.

Altogether our findings lay the foundation for the examination of amino acid requirements in malaria parasites as a versatile therapeutic target for multiple stages (Fig. 8). As malaria parasites are auxotrophic to most of their amino acids^15^,^17^,^26^, depleting their extracellular sources would effectively interfere with the development of sexual stages in mosquitoes and liver stages in vertebrate hosts. The notion of extracellular amino acid depletion has thus far been pursued only in cancer therapies^22^,^23^,^49^, and the present study highlights its relevance to malaria treatment. For amino acids such as asparagine synthesized in the parasite, this approach may be combined with inhibitors specific to parasite enzymes. It would be interesting to examine whether adenylated sulfoximine derivatives (transition-state analogues capable of inhibiting AS and thus capable of suppressing the proliferation of asparaginase-resistant leukaemia cell lines^21^,^24^) may be combined with asparaginase treatment to prevent the sexual- and liver-stage development of *Pb*WT parasites. Furthermore, asparaginase resistance leading to subsequent relapse in patients with ALL has been mainly attributed to the increased expression of AS and to the reduced efflux of asparagine in leukaemia cells^21^,^24^ that are mediated by various signalling events involving amino acid response, survival-related MEK/ERK and mTORC pathways^21^,^50^. However, the existence of a rudimentary amino acid response pathway lacking key homologues of downstream transcriptional factors that control the starvation response together with atypical kinase cascades and the absence of TORC and TORC-associated nutrient-sensing mechanisms in *Plasmodium*^17^,^51^ suggest that the parasite responses in terms of resistance development may be different from those of cancer cells, and this issue will require further investigation.

Transporters that facilitate the uptake of amino acids in malaria parasites need to be explored for new targets. Interestingly, the physiological relevance of transporters in malaria parasites remains unclear, with only one putative amino acid/auxin permease transporter annotated in the parasite genome. It appears that malaria parasites have evolved with a divergent set of transporters for amino acid uptake and that probable candidates may be those belonging to the major facilitator superfamily and ATP-binding cassette superfamily^52^,^53^. Aminoacyl-tRNA synthetases (AaRS) of malaria parasites may serve as another set of targets. As AaRS inhibitors are known for their anti-bacterial and anti-fungal properties, international efforts have been dedicated to the development of new compounds with better efficacy levels^54^,^55^. It has recently been shown that analogues of borrelidin-inhibiting threonyl-tRNA synthetase can offer 100% protection in *P. yoelii*-infected mice^28^. Inhibitors are also available for asparaginyl-tRNA synthetases, of which tirandamycin B from *Streptomyces* sp. 17,944 was shown to exhibit *in vitro* antifilarial activity against *Brugia malayi*, a parasitic nematode that causes elephantiasis^56^. It would be of interest to examine the antimalarial potential of asparaginyl-tRNA synthetase inhibitors in malaria parasites. Thus, a combination of strategies that deplete extracellular amino acids while targeting biosynthetic enzymes, transporters and AaRS in parasites may be explored for the development of a single-exposure radical cure with prophylaxis and chemoprotection^2^,^11^,^12^. Over the last few years, a collective endeavour spearheaded by the Medicines for Malaria Venture (MMV; a not for profit public–private partnership) in collaboration with academic entities and pharmaceutical companies led to the identification of more than 25,000 compounds with submicromolar IC_50_ values via high-throughput phenotypic screening tests performed against asexual stages of the malaria parasite. Approximately 400 compounds were selected based on their drug- and probe-like properties and were made available through the ‘Open Access Malaria Box'^2^,^3^. It would be therefore worthwhile to screen the already existing MMV portfolio for possible leading candidates that may target the aforementioned aspects of asparagine requirement in malaria parasites. To conclude, targeting the asparagine requirement in malaria parasites offers new therapeutic options to combat malaria.

## Methods

### Over-expression and purification of *Pb*AS

Total RNA was isolated from *P. berghei* (*Pb*) ANKA strain (MRA-311, Malaria Research and Reference Reagent Resource Center (MR4), ATCC Manassas Virginia) using TRI reagent (Sigma-Aldrich) according to manufacturer's protocol. For cDNA synthesis, 1 μg of total RNA was used and RT-PCR was carried out with RevertAid Reverse Transcriptase (ThermoFisher Scientific) and Phusion High-Fidelity DNA Polymerase (New England Biolabs) using forward (5′-GCCAGGATCCGATGTGTGGAATTTTAGCTATTTTTCATTCATC-3′) and reverse (5′-GCCCCTCGAGGGCGGCTTTTATATCTTCAATATTTTTTG-3′) primers. The restriction sites are underlined. While designing the reverse primer, the stop codon was omitted to ensure the in-frame alignment of *PbAS* cDNA with the vector-encoded 6xHis-Tag. In brief, RT reaction was performed at 37 °C for 1 h using reverse primer followed by PCR amplification (40 cycles) at 98 °C for 15 s, 55 °C for 15 s and 72 °C for 30 s using forward and reverse primers. *PbAS* cDNA obtained was then digested with *BamHI* and *XhoI* and cloned into *pET-20b(*±) plasmid (Novagen). The recombinant plasmid was transformed into *E. coli* Rosetta2DE3pLysS strain (Novagen) and the cells were grown to an A_600_ of 1.0 at 30 °C, followed by the induction with 1 mM isopropyl-β-D-thiogalactoside for 12 h at 18 °C. The recombinant *Pb*AS was then purified using Ni^2^±-NTA resin (Qiagen). In brief, bacterial cell pellet was resuspended in lysis buffer containing 50 mM Tris pH 8.0, 50 mM NaCl, 20% glycerol, 0.01% Triton X-100 and 1 mM dithiothreitol with protease inhibitors (aprotinin, pepstatin A and leupeptin; 1 μg ml^−1^) and lysed by sonication. The lysate was centrifuged at 50,000 × *g* for 1 h and the supernatant was applied onto a column packed with Ni^2+^-NTA resin. After washing sequentially with lysis buffer containing 1, 10 and 30 mM imidazole, recombinant *Pb*AS was eluted with lysis buffer containing 150 mM imidazole. The protein was then dialyzed against lysis buffer, quantified using Bio-Rad Protein Assay Dye Reagent and stored at −80 °C. The total yield of recombinant *Pb*AS was around 0.3–0.4 mg l^−1^ of bacterial culture with the eluted peak fractions containing protein concentrations of 0.1–0.15 μg μl^−1^. In-solution trypsin digestion for recombinant *Pb*AS was carried out using mass spectrometry grade Trypsin-ultra (New England Biolabs).

### Enzyme assays

*Pb*AS activity was measured as described previously^32^ using a coupled assay system in which inorganic pyrophosphate generated during asparagine synthesis is quantified by monitoring the oxidation of NADH at 340 nm. Briefly, the assays were carried out in a total volume of 1 ml with different concentrations of aspartate in reaction mixtures containing 50 mM Tris pH 8.0, 10 mM ATP, 10 mM MgCl_2_, either 5 mM glutamine or 100 mM NH_4_Cl, 350 μl of pyrophosphate reagent and 3 μg of recombinant *Pb*AS. Radioactive assays were performed in a 25 μl reaction volume containing 20 mM Tris pH 8.0, 20 mM NaCl, 10 mM ATP, 10 mM MgCl_2_, 5 mM glutamine, 0.5 μg of recombinant *Pb*AS and 1 μCi of [U-^14^C]-aspartate (200 mCi mmol^−1^). After incubating at 37 °C for 1 h, the assays were terminated by heating the reaction mixtures at 95 °C for 15 min followed by centrifugation at 15,000 × *g* for 1 min. 10 μl aliquots of the supernatants were then spotted on silica gel TLC sheets. The radiolabelled asparagine was separated using a solvent system of isopropanol and water (7:3) and identified by exposing the TLC sheets to phosphorimager screen for 12 h. To detect AS activity in *P. berghei* parasite lysates, blood collected from infected mice was subjected to saponin lysis and the parasite pellet obtained (∼100 mg) was resuspended in 200 μl of 50 mM Tris pH 8.0 containing 50 mM NaCl, 20% glycerol, 0.01% Triton X-100 and 1 mM DTT, and sonicated. The lysate was then centrifuged at 20,000 × *g* for 20 min to remove the membrane debris and the supernatant obtained was used for assays.

### Generation of *Pb*ASKO parasites

*P. berghei* ANKA genomic DNA was isolated and PCR was carried out to amplify the 5′- and 3′-UTRs of *PbAS*. The primers used were: 5′-UTR (forward)—5′-GCCAGGGCCCAATAATTATGAAGAGATAAAAAATAATTGCAC-3′; 5-′UTR (reverse)—5′-GCCCAGATCTTATTTTATATGCGAATACGTATTTTTTTGC-3′; 3′-UTR (forward)—5′-GCCAGGTACCCACGATTGACATTAACACCATAATTATTCACG-3′; 3′-UTR (reverse)—5′-GCCCGCGGCCGCGTGTATACAACATATATATATCCTCATTTTGC-3′. The amplified 5′- and 3′-UTR products were digested with *ApaI* and *BglII*, and *KpnI* and *NotI*, respectively, and cloned into *pL0006* plasmid (MRA-775, MR4, ATCC Manassas Virginia). The construct was then linearized with *ApaI* and *NotI*, and nucleofected (Nucleofector 2b, Lonza, Switzerland) into mature schizonts of *P. berghei* ANKA, which were injected intravenously into 6-week-old Swiss male mice^57^. The transfected parasites were then recovered using pyrimethamine selection (0.07 mg ml^−1^ in drinking water) and cloned by limiting dilution. Gene deletion in the resulting KO (ASKO) parasites was examined by PCR using *PbAS*-specific primers and confirmed further by Southern, northern and western analyses. For Southern analysis, genomic DNA preparations (10 μg) from *Pb*WT and *Pb*ASKO parasites were subjected to *BsrGI* and *XbaI* digestion followed by hybridization with 5′-UTR-specific probe that was synthesized using Klenow Fragment (New England Biolabs) with 5′-UTR PCR product as a template in the presence of 5 μCi [α-^32^P]-dATP. Northern analysis was carried out with 2 μg of total RNA prepared from *Pb*WT and *Pb*ASKO parasites that were hybridized with *Pb*AS-specific probe synthesized by Klenow Fragment using *PbAS* cDNA as a template. For control, GAPDH-specific probe was synthesized using *PbGAPDH* cDNA obtained with *Pb*GAPDH reverse primer (5′-TTAATTTTTGGTGATGTGGATAGCCAAATC-3′). Western analysis was carried out with the lysates of *Pb*WT and *Pb*ASKO parasites prepared as described for enzyme assays. Anti-AS mouse IgG and anti-hsp60 rabbit IgG (control) were used in 1:1,000 dilutions.

### Parasite maintenance and isolation

*P. berghei* ANKA WT and ASKO parasites were routinely propagated in Swiss male mice (6–8 weeks old) by injecting 10^5^ parasitized RBCs/reticulocytes intraperitoneally^58^,^59^. Giemsa-stained thin smears for blood collected from tail vein were prepared to quantify the percentage of parasitemia and assess the parasite growth. When the parasitemia was around 10%, mice were anaesthetised using ketamine/xylazine and the infected blood was collected through cardiac puncture. After quantifying the parasitemia and counting the RBCs/reticulocytes using a hemocytometer, the infected blood was diluted with PBS to initiate fresh infections in naive mice. *P. berghei* parasites were isolated by treating the infected erythrocytes with an equal volume of 0.15% (w/v) saponin in PBS followed by centrifugation at 10,000 × *g* for 10 min^60^. The parasite pellet was then washed four times with ice-cold PBS to remove haemoglobin and other red blood cell contaminants.

### Maintenance of *A. stephensi* mosquitoes

The rearing of *A. stephensi* mosquitoes (*stephensi* sensu stricto, National Institute of Malaria Research, Bangalore) was carried out under standard insectary conditions maintained at 27 °C and 75–80% humidity with a 12 h light and dark photo-period as described earlier^61^,^62^. Eggs were produced by blood feeding the adult female mosquitoes on Swiss male mice (6–8 weeks old) anaesthetised with ketamine/xylazine. Larvae were reared using standard procedures and the pupae were segregated into cages for adult emergence. The adult mosquitoes were fed with 10% glucose solution containing 0.05% paraminobenzoic acid (feeding solution).

### *P. berghei* infection studies in *A. stephensi* mosquitoes

To perform sexual stage studies, 5–7 days old adult female mosquitoes were allowed to feed on Swiss male mice (6–8 weeks old) infected with *P. berghei* WT or ASKO parasites. Mosquito feeding experiments were carried out on day 8 post infection when the blood parasitemia was around 10% with at least two exflagellation centres per field. The fully engorged mosquitoes were then segregated and maintained at 19 °C with 75–80% humidity. The mosquito midguts were dissected at 20 h post feeding to remove the blood bolus and quantify the number of ookinetes formed^63^. To determine the number of oocysts, mosquito midguts were dissected on day 10 post feeding and mercurochrome staining was carried out as described^64^. For sporozoite analyses, the salivary glands were dissected on day 19 and the sporozoites were extracted and counted using hemocytometer^65^. Asparagine supplementation to mosquitoes was carried out in feeding solution containing 0.5% (w/v) asparagine. The supplementation was started immediately after blood feeding and continued till the sporozoites were collected. To initiate the liver infections, 10^4^ sporozoites were injected intravenously into naive Swiss male mice (6–8 weeks old). The appearance of asexual stages and their subsequent development were monitored by examining the Giemsa-stained blood smears.

### Asparaginase treatment and asparagine supplementation

Asparaginase treatment was carried out in uninfected mice or mice infected with *Pb*WT or *Pb*ASKO parasites by injecting each mouse with 50 IU of *E. coli* L-asparaginase dissolved in saline (Dr Reddy's Laboratories Ltd., India) through intramuscular route. For exflagellation analyses, mice were treated with three doses of asparaginase on day 5, 6 and 7. For liver-stage analyses, mice were treated with three doses of asparaginase on day 0, 1 and 2 followed by sporozoite injection on day 1. For asparagine supplementation studies, asparaginase treatment was reduced to two doses. Asparagine supplementation in mice was carried out at 12 h interval by injecting 200 μl of asparagine solution (30 mg ml^−1^ in water) through intraperitoneal route and the restoration of exflagellation was analysed on day 8.

### Quantification of plasma asparagine levels

To quantify plasma asparagine levels, blood samples collected from the peri-orbital sinus of mice using microhematocrit capillary tubes were centrifuged at 2,000 × *g* for 15 min, 4 °C. The supernatant obtained was again centrifuged at 17,000 × *g* for 5 min to remove any residual cells or cell debris and used immediately for asparagine quantification. In brief, the plasma samples were precipitated with acetone and centrifuged at 15,000 × *g* for 10 min at 4 °C to remove proteins. The supernatant obtained was derivatized with 6-aminoquinolyl-N-hydroxysuccinimidyl carbamate^66^ and the metabolites were extracted using reversed phase-solid phase extraction cartridges. The eluant was then dried and reconstituted in 0.5% (v/v) acetonitrile followed by loading onto Agilent SB—C18 (100 × 2.1 mm) column. The HPLC run was carried out using 10 mM ammonium acetate and acetonitrile solvent system. Mass spectrometry analysis was performed using Thermo fisher-TSQ vantage mass spectrometer and the conditions followed are as follows: spray voltage, 4,000 V; ion transfer capillary temperature, 280 °C; source temperature, 100 °C; sheath gas and auxiliary gas, 20 and 10, respectively (arbitrary units); collision gas, argon; scan time, 50 ms per transition with ion polarity positive and S-lens voltage optimized for individual metabolites. Hydroxyproline-D3 was used as an internal standard (CDN isotope, Quebec, Canada). The final quantification of asparagine in mouse plasma samples was done based on the standard curve generated using different concentrations of asparagine (Sigma-Aldrich).

### Immunoflourescence analyses of EEFs

To analyse the formation of EEFs, mice treated with and without asparaginase were infected intravenously with 10^5^ *Pb*WT or *Pb*ASKO sporozites. After 50 h post-infection, hepatocytes from infected mice were isolated as described earlier^67^ and thin smears were prepared. The smears were fixed with cold acetone for 10 min at −20 °C, blocked subsequently at room temperature for 3 h with PBS containing 3% (w/v) bovine serum albumin followed by 3 h incubation with polyclonal anti-hsp70 rabbit serum (1:50 dilution)^68^. After washing with PBS, the hepatocytes were treated for 2 h with FITC-conjugated donkey anti-rabbit secondary antibodies (Santa Cruz; 1:100 dilution). DAPI (1 μg ml^−1^ in PBS) staining was carried out for 10 min and immunofluorescence images of hepatocytes mounted with Fluoroshield (Sigma-Aldrich) were captured using 100 × objective. For quantification of EEFs, hepatocytes isolated from three different mice were pooled and the average number of hepatocytes per field was calculated from 20 different fields to estimate hsp70-positive EEFs present in total number of fields corresponding to 10^4^ hepatocytes and 30 such independent readings were used to plot the graphs.

### Exflagellation analyses of male gametocytes

For exflagellation analyses, approximately 5–10 μl of blood collected from tail vein of infected mice was diluted with 1 ml of exflagellation medium and used immediately to count the exflagellation centres as described earlier^69^. To ensure the absence of exflagellation, a minimum of 50 fields were examined over a period of 45 min for the blood collected from each mouse. This was also further confirmed by performing blood-feeding studies in mosquitoes. Live imaging of male gametocyte exflagellation was carried out under bright field with 60 × objective using Leica differential interference contrast microscope. The images were captured at 300 time points for an interval of every 20 ms with 2 × 2 binning. To generate videos, the images were processed using ImageJ software.

### Other procedures

Southern, northern and western analyses were carried out using standard protocols. Exflagellation medium was prepared as described earlier^69^. To generate polyclonal serum, mice were immunized with recombinant protein emulsified in Freunds adjuvant followed by three boosters. IgG purification was carried out using Protein A/G agarose beads.

### Statistical analyses

*P* values were determined using unpaired Student's *t*-test of Excel software performed for two-tailed distribution and unequal sample variance. *P*<0.05 was considered as significant. To plot the graphs and perform regression analyses, SigmaPlot software version 10 was used. Error bars given in figures represent the s.d.

### Ethics statement

*P. berghei* infection studies in mosquitoes and mice were carried out as per the guidelines of the Committee for the Purpose and Supervision of Experimental animals (CPCSEA), Government of India (Registration No: 48/1999/CPCSEA) and as approved by the Institutional Animal Ethics Committee of the Indian Institute of Science, Bangalore (CAF/Ethics/319/2013).

## Additional information

**How to cite this article:** Nagaraj, V. A. *et al*. Asparagine requirement in *Plasmodium berghei* as a target to prevent malaria transmission and liver infections. *Nat. Commun.* 6:8775 doi: 10.1038/ncomms9775 (2015).

## Supplementary Material

Supplementary Information Supplementary Figures 1-6

Supplementary Movie 1 Exflagellation of *Pb*WT male gametocytes. Blood collected from *Pb*WT-infected mice was diluted with exflagellation medium and used for exflagellation analysis. Exflagellating male gametocyte can be easily identified as a wriggling cell with attached flagella.

Supplementary Movie 2 Exflagellation of *Pb*ASKO male gametocytes. Blood collected from *Pb*ASKO-infected mice was diluted with exflagellation medium and used for exflagellation analysis.

Supplementary Movie 3 Exflagellation of asparaginase-treated *Pb*ASKO male gametocytes. Mice were treated with three doses of asparaginase. On day 8, blood collected from asparaginase-treated *Pb*ASKO-infected mice was diluted with exflagellation medium and used. Video given here is for the purpose of representation. The absence of exflagellation was confirmed by examining multiple fields over a period of 45 min. This was further validated by ensuring the absence of sexual stage development in mosquitoes.

Supplementary Movie 4 Exflagellation of asparaginase-treated *Pb*WT male gametocytes. Mice were treated with three doses of asparaginase. On day 8, blood collected from asparaginase-treated *Pb*WT-infected mice was diluted with exflagellation medium and used.

Supplementary Movie 5 Restoration of *Pb*ASKO male gametocyte exflagellation by *in vivo* asparagine supplementation. Mice treated with two doses of asparaginase were subjected to *in vivo* supplementation of asparagine. Exflagellation analysis was carried out on day 8 by diluting the blood with exflagellation medium.

## References

[b1] Global Report on Antimalarial Drug Efficacy and Drug Resistance: 2000–2010. World Health Organization, Geneva, Switzerland. Available at http://apps.who.int/iris/bitstream/10665/44449/1/9789241500470_eng.pdf.

[b2] Wells T. N., van Huijsduijnen R. H. & Van Voorhis W. C. Malaria medicines: a glass half full? Nat. Rev. Drug Discov. 14, 424–442 (2015). 26000721 10.1038/nrd4573

[b3] Flannery E. L., Chatterjee A. K. & Winzeler E. A. Antimalarial drug discovery - approaches and progress towards new medicines. Nat. Rev. Microbiol. 11, 849–862 (2013). 24217412 10.1038/nrmicro3138 PMC3941073

[b4] Dondorp A. M. . Artemisinin resistance in *Plasmodium falciparum* malaria. N. Engl. J. Med. 361, 455–467 (2009). 19641202 10.1056/NEJMoa0808859 PMC3495232

[b5] Dondorp A. M. . Artemisinin resiatnace: current status and scenarios for containment. Nat. Rev. Microbiol. 8, 272–280 (2010). 20208550 10.1038/nrmicro2331

[b6] Greenwood B. Treatment of malaria - a continuing Challenge. N. Engl. J. Med. 371, 474–475 (2014). 25075840 10.1056/NEJMe1407026

[b7] Tun K. M. . Spread of artemisinin-resistant *Plasmodium falciparum* in Myanmar: a cross-sectional survey of the K13 molecular marker. Lancet Infect. Dis. 15, 415–421 (2015). 25704894 10.1016/S1473-3099(15)70032-0 PMC4374103

[b8] Mungthin M., Bray P. G., Ridley R. G. & Ward S. A. Central role of hemoglobin degradation in mechanisms of action of 4-aminoquinolines, quinoline methanols, and phenanthrene methanols. Antimicrob. Agents Chemother. 42, 2973–2977 (1998). 9797235 10.1128/aac.42.11.2973 PMC105975

[b9] Klonis N. . Artemisinin activity against *Plasmodium falciparum* requires hemoglobin uptake and digestion. Proc. Natl Acad. Sci. USA 108, 11405–11410 (2011). 21709259 10.1073/pnas.1104063108 PMC3136263

[b10] Coronado L. M., Nadovich C. T. & Spadafora C. Malarial hemozoin: from target to tool. Biochim. Biophys. Acta 1840, 2032–2041 (2014). 24556123 10.1016/j.bbagen.2014.02.009 PMC4049529

[b11] Burrows J. N., van Huijsduijnen R. H., Möhrle J. J., Oeuvray C. & Wells T. N. Designing the next generation of medicines for malaria control and eradication. Malar. J. 12, 187 (2013). 23742293 10.1186/1475-2875-12-187 PMC3685552

[b12] Vial H. . CRIMALDDI: platform technologies and novel anti-malarial drug targets. Malar. J. 12, 396 (2013). 24498961 10.1186/1475-2875-12-396 PMC3827883

[b13] Wu G. Functional amino acids in growth, reproduction, and health. Adv. Nutr. 1, 31–37 (2010). 22043449 10.3945/an.110.1008 PMC3042786

[b14] Wu G. Amino acids: metabolism, functions, and nutrition. Amino Acids 37, 1–17 (2009). 19301095 10.1007/s00726-009-0269-0

[b15] Gardner M. J. . Genome sequence of the human malaria parasite *Plasmodium falciparum*. Nature 419, 498–511 (2002). 12368864 10.1038/nature01097 PMC3836256

[b16] Payne S. H. & Loomis W. F. Retention and loss of amino acid biosynthetic pathways based on analysis of whole-genome sequences. Eukaryot. Cell 5, 272–276 (2006). 16467468 10.1128/EC.5.2.272-276.2006 PMC1405893

[b17] Babbitt S. E. . *Plasmodium falciparum* responds to amino acid starvation by entering into a hibernatory state. Proc. Natl Acad. Sci. USA 109, E3278–E3287 (2012). 23112171 10.1073/pnas.1209823109 PMC3511138

[b18] Yadav M. K. & Swati D. Comparative genome analysis of six malarial parasites using codon usage bias based tools. Bioinformation 8, 1230–1239 (2012). 23275725 10.6026/97320630081230 PMC3530877

[b19] Filisetti D. . Aminoacylation of *Plasmodium falciparum* tRNA(Asn) and insights in the synthesis of asparagine repeats. J. Biol. Chem. 288, 36361–36371 (2013). 24196969 10.1074/jbc.M113.522896 PMC3868750

[b20] Dalby A. R. A comparative proteomic analysis of the simple amino acid repeat distributions in *Plasmodia* reveals lineage specific amino acid selection. PLoS ONE 4, e6231 (2009). 19597555 10.1371/journal.pone.0006231 PMC2705789

[b21] Richards N. G. & Kilberg M. S. Asparagine synthetase chemotherapy. Annu. Rev. Biochem. 75, 629–654 (2006). 16756505 10.1146/annurev.biochem.75.103004.142520 PMC3587692

[b22] Pieters R. . L-asparaginase treatment in acute lymphoblastic leukemia: a focus on *Erwinia* asparaginase. Cancer 117, 238–249 (2011). 20824725 10.1002/cncr.25489 PMC3000881

[b23] Avramis V. I. & Tiwari P. N. Asparaginase (native ASNase or pegylated ASNase) in the treatment of acute lymphoblastic leukemia. Int. J. Nanomed. 1, 241–254 (2006). PMC2426805 17717965

[b24] Ikeuchi H. . A sulfoximine-based inhibitor of human asparagine synthetase kills L-asparaginase-resistant leukemia cells. Bioorg. Med. Chem. 20, 5915–5927 (2012). 22951255 10.1016/j.bmc.2012.07.047

[b25] Salcedo-Sora J. E., Caamano-Gutierrez E., Ward S. A. & Biagini G. A. The proliferating cell hypothesis: a metabolic framework for *Plasmodium* growth and development. Trends Parasitol. 30, 170–175 (2014). 24636355 10.1016/j.pt.2014.02.001 PMC3989997

[b26] Liu J., Istvan E. S., Gluzman I. Y., Gross J. & Goldberg D. E. *Plasmodium falciparum* ensures its amino acid supply with multiple acquisition pathways and redundant proteolytic enzyme systems. Proc. Natl Acad. Sci. USA 103, 8840–8845 (2006). 16731623 10.1073/pnas.0601876103 PMC1470969

[b27] Istvan E. S. . Validation of isoleucine utilization targets in *Plasmodium falciparum*. Proc. Natl Acad. Sci. USA 108, 1627–1632 (2011). 21205898 10.1073/pnas.1011560108 PMC3029723

[b28] Novoa E. M. . Analogs of natural aminoacyl-tRNA synthetase inhibitors clear malaria *in vivo*. Proc. Natl Acad. Sci. USA 111, E5508–E5517 (2014). 25489076 10.1073/pnas.1405994111 PMC4280603

[b29] Cedar H. & Schwartz J. H. The asparagine synthetase of *Escherichia coli*. II. Studies on mechanism. J. Biol. Chem. 244, 4122–4127 (1969). 4895362

[b30] Van Heeke G. & Schuster S. M. Expression of human asparagine synthetase in *Escherichia coli*. J. Biol. Chem. 264, 5503–5509 (1989). 2564390

[b31] Larsen T. M. . Three-dimensional structure of *Escherichia coli* asparagine synthetase B: a short journey from substrate to product. Biochemistry 38, 16146–16157 (1999). 10587437 10.1021/bi9915768

[b32] Ciustea M., Gutierrez J. A., Abbatiello S. E., Eyler J. R. & Richards N. G. Efficient expression, purification, and characterization of C-terminally tagged, recombinant human asparagine synthetase. Arch. Biochem. Biophys. 440, 18–27 (2005). 16023613 10.1016/j.abb.2005.05.023

[b33] Waters A. P., Thomas A. W., van Dijk M. R. & Janse C. J. Transfection of malaria parasites. Methods 13, 134–147 (1997). 9405197 10.1006/meth.1997.0506

[b34] Singh G. P. . Hyper-expansion of asparagines correlates with an abundance of proteins with prion-like domains in *Plasmodium falciparum*. Mol. Biochem. Parasitol. 137, 307–319 (2004). 15383301 10.1016/j.molbiopara.2004.05.016

[b35] Zilversmit M. M. . Low-complexity regions in *Plasmodium falciparum*: missing links in the evolution of an extreme genome. Mol. Biol. Evol. 27, 2198–2209 (2010). 20427419 10.1093/molbev/msq108 PMC2922621

[b36] Bogatyreva N. S., Finkelstein A. V. & Galzitskaya O. V. Trend of amino acid composition of proteins of different taxa. J. Bioinform. Comput. Biol. 4, 597–608 (2006). 16819805 10.1142/s0219720006002016

[b37] Muralidharan V., Oksman A., Iwamoto M., Wandless T. J. & Goldberg D. E. Asparagine repeat function in a *Plasmodium falciparum* protein assessed via a regulatable fluorescent affinity tag. Proc. Natl Acad. Sci. USA 108, 4411–4416 (2011). 21368162 10.1073/pnas.1018449108 PMC3060247

[b38] Muralidharan V. & Goldberg D. E. Asparagine repeats in *Plasmodium falciparum* proteins: good for nothing? PLoS Pathog. 9, e1003488 (2013). 23990777 10.1371/journal.ppat.1003488 PMC3749963

[b39] Reitzer L. J. & Magasanik B. Asparagine synthetases of *Klebsiella aerogenes*: properties and regulation of synthesis. J. Bacteriol. 151, 1299–1313 (1982). 6125499 10.1128/jb.151.3.1299-1313.1982 PMC220408

[b40] Blaise M. . Crystal structure of the archaeal asparagine synthetase: interrelation with aspartyl-tRNA and asparaginyl-tRNA synthetases. J. Mol. Biol. 412, 437–452 (2011). 21820443 10.1016/j.jmb.2011.07.050

[b41] Gowri V. S., Ghosh I., Sharma A. & Madhubala R. Unusual domain architecture of aminoacyl tRNA synthetases and their paralogs from *Leishmania major*. BMC Genomics 13, 621 (2012). 23151081 10.1186/1471-2164-13-621 PMC3532385

[b42] Ramos F. & Wiame J. M. Two asparagine synthetases in *Saccharomyces cerevisiae*. Eur. J. Biochem. 108, 373–377 (1980). 6105958 10.1111/j.1432-1033.1980.tb04732.x

[b43] Duff S. M. . A kinetic comparison of asparagine synthetase isozymes from higher plants. Plant Physiol. Biochem. 49, 251–256 (2011). 21276727 10.1016/j.plaphy.2010.12.006

[b44] Aly A. S., Vaughan A. M. & Kappe S. H. Malaria parasite development in the mosquito and infection of the mammalian host. Annu. Rev. Microbiol. 63, 195–221 (2009). 19575563 10.1146/annurev.micro.091208.073403 PMC2841446

[b45] Rytting M. E. Role of L-asparaginase in acute lymphoblastic leukemia: focus on adult patients. Blood Lymphat. Cancer 2, 117–124 2012.

[b46] Mair G. R. . Regulation of sexual development of *Plasmodium* by translational repression. Science 313, 667–669 (2006). 16888139 10.1126/science.1125129 PMC1609190

[b47] Raabe A. C., Billker O., Vial H. J. & Wengelnik K. Quantitative assessment of DNA replication to monitor microgametogenesis in *Plasmodium berghei*. Mol. Biochem. Parasitol. 168, 172–176 (2009). 19712704 10.1016/j.molbiopara.2009.08.004 PMC2789244

[b48] Baker D. A. Malaria gametocytogenesis. Mol. Biochem. Parasitol. 172, 57–65 (2010). 20381542 10.1016/j.molbiopara.2010.03.019 PMC2880792

[b49] Cantor J. R., Panayiotou V., Agnello G., Georgiou G. & Stone E. M. Engineering reduced-immunogenicity enzymes for amino acid depletion therapy in cancer. Methods Enzymol. 502, 291–319 (2012). 22208990 10.1016/B978-0-12-416039-2.00015-X

[b50] Bunpo P. . GCN2 protein kinase is required to activate amino acid deprivation responses in mice treated with the anti-cancer agent L-asparaginase. J. Biol. Chem. 284, 32742–32749 (2009). 19783659 10.1074/jbc.M109.047910 PMC2781691

[b51] Leroy D. & Doerig C. Drugging the *Plasmodium* kinome: the benefits of academia-industry synergy. Trends Pharmacol. Sci. 29, 241–249 (2008). 18394721 10.1016/j.tips.2008.02.005

[b52] Staines H. M. . Exploiting the therapeutic potential of *Plasmodium falciparum* solute transporters. Trends Parasitol. 26, 284–296 (2010). 20392668 10.1016/j.pt.2010.03.004

[b53] Dean P., Major P., Nakjang S., Hirt R. P. & Embley T. M. Transport proteins of parasitic protists and their role in nutrient salvage. Front. Plant Sci. 5, 153 (2014). 24808897 10.3389/fpls.2014.00153 PMC4010794

[b54] Hurdle J. G., O'Neill A. J. & Chopra I. Prospects for aminoacyl-tRNA synthetase inhibitors as new antimicrobial agents. Antimicrob. Agents Chemother. 49, 4821–4833 (2005). 16304142 10.1128/AAC.49.12.4821-4833.2005 PMC1315952

[b55] Lv P. C. & Zhu H. L. Aminoacyl-tRNA synthetase inhibitors as potent antibacterials. Curr. Med. Chem. 19, 3550–3563 (2012). 22680640 10.2174/092986712801323199

[b56] Rateb M. E. . Medium optimization of Streptomyces sp. 17944 for tirandamycin B production and isolation and structural elucidation of tirandamycins H, I and J. J. Antibiot. (Tokyo) 67, 127–132 (2014). 23715040 10.1038/ja.2013.50 PMC3773001

[b57] Janse C. J., Ramesar J. & Waters A. P. High-efficiency transfection and drug selection of genetically transformed blood stages of the rodent malaria parasite *Plasmodium berghei*. Nat. Protoc. 1, 346–356 (2006). 17406255 10.1038/nprot.2006.53

[b58] Cox F. E. G. in Malaria: Principles and Practice of Malariology (eds Wernsdorfer W. H., McGregor I. 1503–1543Edinburgh, Churchill Livingstone (1988).

[b59] Helmby H. & de souza B. in Methods in Malaria Research eds Moll K., Ljungstrom I., Perlmann H., Scherf A., Wahlgren M. 147–148MR4; ATCC: BioMalPar, Paris, France (2008).

[b60] Cowman A. E., Karcz S., Galatis D. & Culvenor J. G. A P-glycoprotein homologue of *Plasmodium falciparum* is localized on the digestive vacuole. J. Cell. Biol. 113, 1033–1042 (1991). 1674943 10.1083/jcb.113.5.1033 PMC2289011

[b61] Benedict M. Q. in The Molecular Biology of Disease Vectors: A Methods Manual eds Crampton J. M., Beard C. B., Louis C. 3–12Champman and Hall (1997).

[b62] MR4 staff. in *Methods in Anopheles Research*, 58–91 (MR4; ATCC; National Institutes of Health; Centres for Disease Control and Prevention, 2010).

[b63] Shimizu S., Osada Y., Kanazawa T., Tanaka Y. & Arai M. Suppressive effect of azithromycin on *Plasmodium berghei* mosquito stage development and apicoplast replication. Malar. J. 9, 73 (2010). 20219090 10.1186/1475-2875-9-73 PMC2846956

[b64] Usui M., Fukumoto S., Inoue N. & Kawazu S. Improvement of the observational method for *Plasmodium berghei* oocysts in the midgut of mosquitoes. Parasit. Vectors 4, 118 (2011). 21707972 10.1186/1756-3305-4-118 PMC3141744

[b65] Touray M. G., Warburg A., Laughinghouse A., Krettli A. U. & Miller L. H. Developmentally regulated infectivity of malaria sporozoites for mosquito salivary glands and the vertebrate host. J. Exp. Med. 175, 1607–1612 (1992). 1588284 10.1084/jem.175.6.1607 PMC2119265

[b66] Sharma G. . Analysis of 26 amino acids in human plasma by HPLC using AQC as derivatizing agent and its application in metabolic laboratory. Amino Acids 46, 1253–1263 (2014). 24515597 10.1007/s00726-014-1682-6

[b67] Tarun A. S. . Quantitative isolation and *in vivo* imaging of malaria parasite liver stages. Int. J. Parasitol. 36, 1283–1293 (2006). 16890231 10.1016/j.ijpara.2006.06.009

[b68] Banumathy G., Singh V. & Tatu U. Host chaperones are recruited in membrane-bound complexes by *Plasmodium falciparum*. J. Biol. Chem. 277, 3902–3912 (2002). 11711553 10.1074/jbc.M110513200

[b69] Guttery D. S. . A Unique Protein Phosphatase With Kelch-Like Domains (PPKL) in Plasmodium modulates ookinete differentiation, motility and invasion. PLoS Pathog. 8, e1002948 (2012). 23028336 10.1371/journal.ppat.1002948 PMC3447748

