# Supplementary material for: Asparagine requirement in Plasmodium berghei as a target to prevent malaria transmission and liver infections
Source: Nat Commun. 2015 Nov 4;6:8775. doi: 10.1038/ncomms9775 (PMC4659947; doi:10.1038/ncomms9775)
Supplement: Supplementary Information — Supplementary Figures 1-6 [file ncomms9775-s1.pdf]

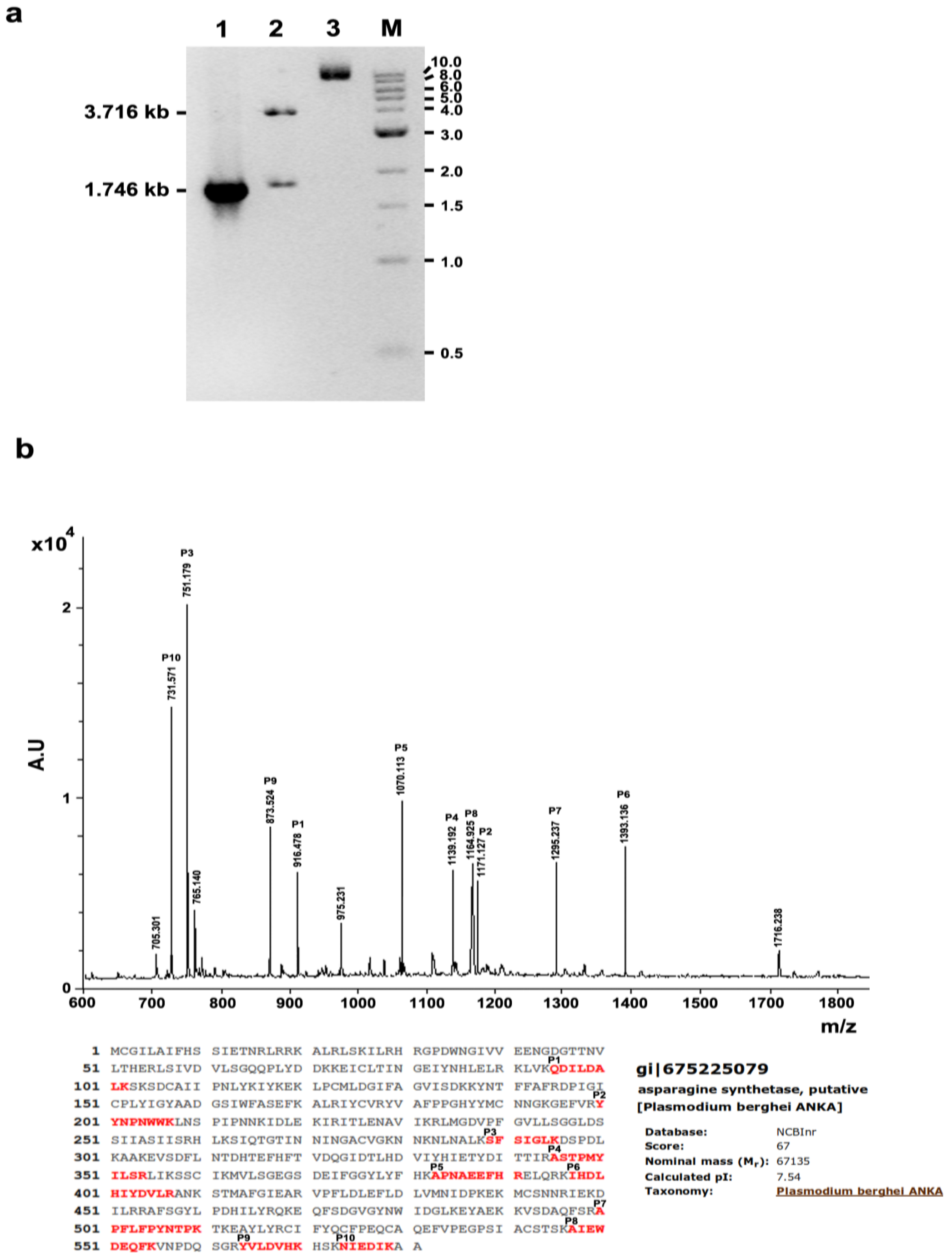

Supplementary Figure 1. Cloning of *Pb*AS cDNA and mass spectrometric analysis of recombinant *Pb*AS. (**a**) Cloning of *PbAS* cDNA into *pET-20b(+)*. Lane 1: *PbAS* cDNA product; Lane 2: *BamHI* and *XhoI* digestion of *pET-20b(+)-PbAS* plasmid showing the release of insert; Lane 3: Undigested *pET-20b(+)-PbAS* plasmid; Lane M: 1 kb DNA ladder (kb). (**b**) MALDI analysis of tryptic peptides generated from recombinant *Pb*AS. The peptide mass fingerprint was searched against the NCBI-nr databse using MASCOT Server. The matched peptides are highlighted in red and labelled as P1-P10.

```
P. berghei         1 MCGILAIFHSSIETNRLRRKALRLSKILRHRGPDWNGIVVEENGDGTTNV     50
                     |||||||||||||.:|||||||.||||||||||||||||||||.||||||
P. falciparum      1 MCGILAIFHSSIEKHRLRRKALNLSKILRHRGPDWNGIVVEENDDGTTNV     50

P. berghei        51 LTHERLSIVDVLSGQQPLYDDKKEICLTINGEIYNHLELRKLVKQDILDA    100
                     |.||||:|||||||.||||||::|:|||||||||||||||||:|::.|:.
P. falciparum     51 LAHERLAIVDVLSGHQPLYDDEEEVCLTINGEIYNHLELRKLIKEENLNK    100

P. berghei       101 LKSKSDCAIIPNLYKIYKEKLPCMLDGIFAGVISDKKYNTFFAFRDPIGI    150
                     |||.||||:||||:||||||:|.||||||||||||||.||||||||||||
P. falciparum    101 LKSCSDCAVIPNLFKIYKEKIPSMLDGIFAGVISDKKNNTFFAFRDPIGI    150

P. berghei       151 CPLYIGYAADGSIWFASEFKALRIYCVRYVAFPPGHYYMCNNGKGEFVRY    200
                     |||||||||||||||:||||||:..|:|||.|||||||..|..|||||||
P. falciparum    151 CPLYIGYAADGSIWFSSEFKALKDNCIRYVIFPPGHYYKNNKNKGEFVRY    200

P. berghei       201 YNPNWWKLNSPIPNNKIDLEKIRITLENAVIKRLMGDVPFGVLLSGGLDS    250
                     ||||||.||:.|||||:|..:|||.||.|||||||||||||:||||||||
P. falciparum    201 YNPNWWSLNNSIPNNKVDFNEIRIHLEKAVIKRLMGDVPFGILLSGGLDS    250

P. berghei       251 SIIASIISRHL--------KSIQTG----TINNINGACVGKNNKNLN---    285
                     ||||:|:::||        ||||.|    :.||.|.:....||.|.|
P. falciparum    251 SIIAAILAKHLNILDNKNGKSIQNGNDKKSNNNGNESNNNNNNNNFNNNN    300

P. berghei       286 ----ALKSFSIGLKDSPDLKAAKEVSDFLNTDHTEFHFTVDQGIDTLHDV    331
                         .|:|||||||.||||||||||:::|..:||||:|||::|||:||||
P. falciparum    301 SGPQKLRSFSIGLKGSPDLKAAKEVAEYLGIEHTEFYFTVEEGIDSLHDV    350

P. berghei       332 IYHIETYDITTIRASTPMYILSRLIKSSCIKMVLSGEGSDEIFGGYLYFH    381
                     |||||||||||||||||||||||||||||:||||||||||||||||||||
P. falciparum    351 IYHIETYDITTIRASTPMYILSRLIKSSCVKMVLSGEGSDEIFGGYLYFH    400

P. berghei       382 KAPNAEEFHRELQRKIHDLHIYDVLRANKSTMAFGIEARVPFLDLEFLDL    431
                     ||||.|||||||||||||||.|||||||||||||||||||||||::||::
P. falciparum    401 KAPNKEEFHRELQRKIHDLHYYDVLRANKSTMAFGIEARVPFLDIQFLNV    450

P. berghei       432 VMNIDPKEKMCSNNRIEKDILRRAFSGYLPDHILYRQKEQFSDGVGYNWI    481
                     ||||||::||||||:|||.|||:||.||||:|||||||||||||||||||
P. falciparum    451 VMNIDPQDKMCSNNKIEKYILRKAFEGYLPEHILYRQKEQFSDGVGYNWI    500

P. berghei       482 DGLKEYAEKKVSDAQFSRAPFLFPYNTPKTKEAYLYRCIFYQCFPEQCAQ    531
                     ||||:|||||:||.|||||.||||||||||||.|||||||.:||||||||
P. falciparum    501 DGLKQYAEKKISDIQFSRAKFLFPYNTPKTKEGYLYRCIFSECFPEQCAQ    550

P. berghei       532 EFVPEGPSIACSTSKAIEWDEQFKVNPDQSGRYVLDVHKHSKNIEDIKAA    581
                     |.||:|.|||||||||:||||.||.|.|||||.||.:|:|||..:|:|..
P. falciparum    551 ESVPQGESIACSTSKAVEWDESFKQNADQSGRSVLGIHRHSKQFDDVKCI    600

P. berghei       582 ----------     581

P. falciparum    601 PLQNQESINY     610
```

Supplementary Figure 2. Sequence alignment of *Pb*AS with *Pf*AS. Pairwise sequence alignment of *Pb*AS and *Pf*AS was performed using the "needle" program (Needleman-Wunsch global Alignment algorithm) of EMBOSS suite.

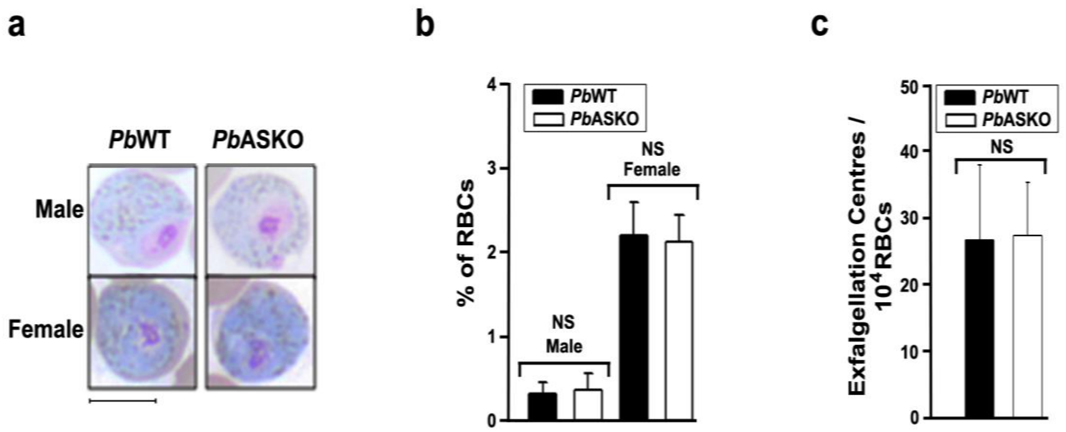

Supplementary Figure 3. Examination of *Pb*WT and *Pb*ASKO gametocytes. (**a**) Bright field images for male (micro) and female (macro) gametocytes of *Pb*WT and *Pb*ASKO stained with Giemsa reagent. Scale bar: 5 µm. (**b**) Quantification of gametocytes. Blood smears were prepared on day 8 post infection and stained with Giemsa reagent. Based on morphology, male and female gamaetocytes were identified and counted. (**c**) Exflagellation centres observed in *Pb*WT and *Pb*ASKO parasites. The Mean ± S.D values were obtained from nine mice. NS, not significant (two-tailed unpaired Student’s *t*-test).

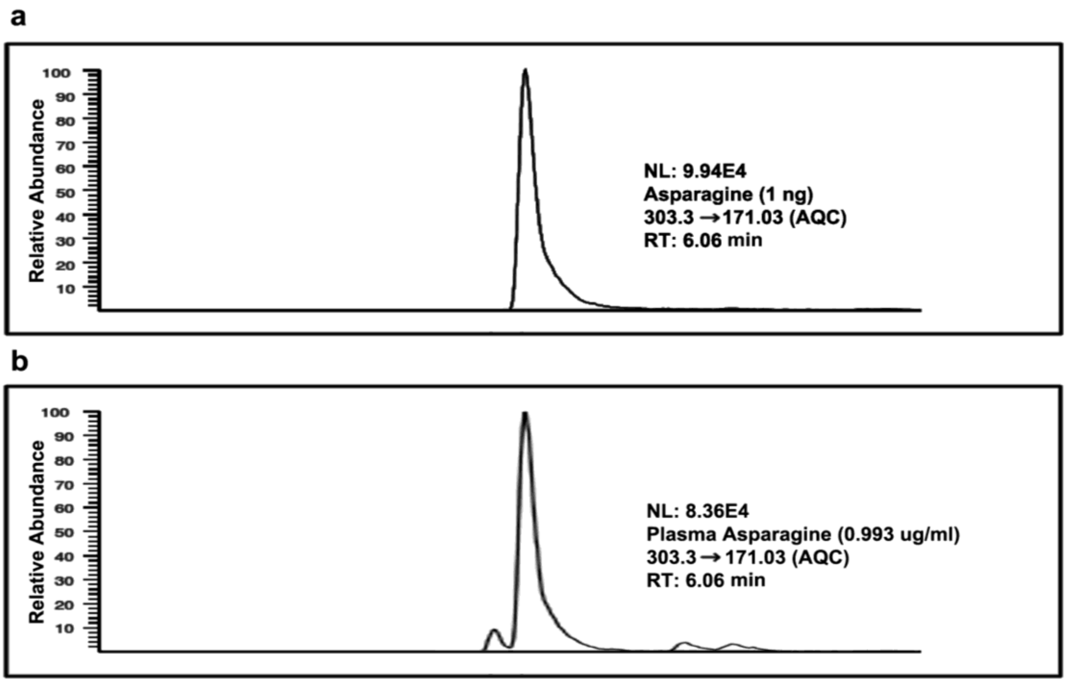

Supplementary Figure 4. UHPLC-MS/SRM based quantification of asparagine in mouse plasma. (**a**) Chromatogram of standard asparagine derivatized with AQC. (**b**) Chromatogram of asparagine present in mouse plasma derivatized with AQC. The plasma samples were prepared as described in methods. Mass spectrometry analysis revealed the presence of parent ion (303.3 m/z) corresponding to derivatized asparagine which upon fragmention produced daughter ion of AQC (171.03 m/z). AQC - 6-aminoquinolyl-N-hydroxysuccinimidyl carbamate; NL - normalized intensity level; RT - retention time.

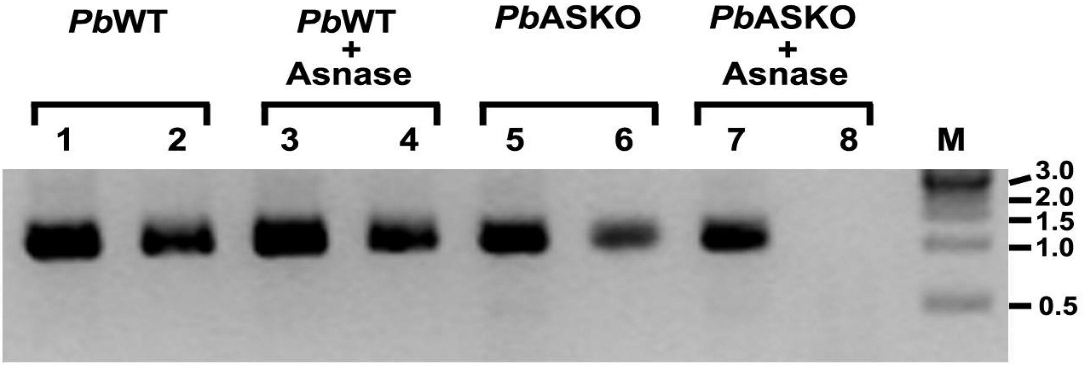

Supplementary Figure 5. RT-PCR analysis of RNA isolated from *Pb*WT and *Pb*ASKO sporozoite-infected mouse liver samples with and without asparaginase treatment. Total RNA (50 h post-infection) was isolated from infected mouse liver samples weighing approximately 150 mg. RNA isolated from three different mice were pooled and cDNA preparation was carried out using 5 µg of total RNA. Lane 1, 3, 5 and 7: RT-PCR with mouse GAPDH specific primers (control; 1 kb); Lane 2, 4, 6 and 8: RT-PCR with *Pb*GAPDH specific primers (1.02 kb); Lane M: DNA ladder (kb). Mouse and parasite GAPDH amplifications were carried out for 30 and 40 cycles, respectively. The primers used were: *Pb*GAPDH (forward): 5’-ATGGCAATAACAAAAGTCGGAATTAATGG-3’; *Pb*GAPDH (reverse): 5’-TTAATTTTTGGTGATGTGGATAGCCAAATC-3’; Mouse GAPDH (forward): 5'-ATGGTGAAGGTCGGTGTGAACGGA-3’; Mouse GAPDH (reverse): 5'-TTACTCCTTGGAGGCCATGTAGG-3'.

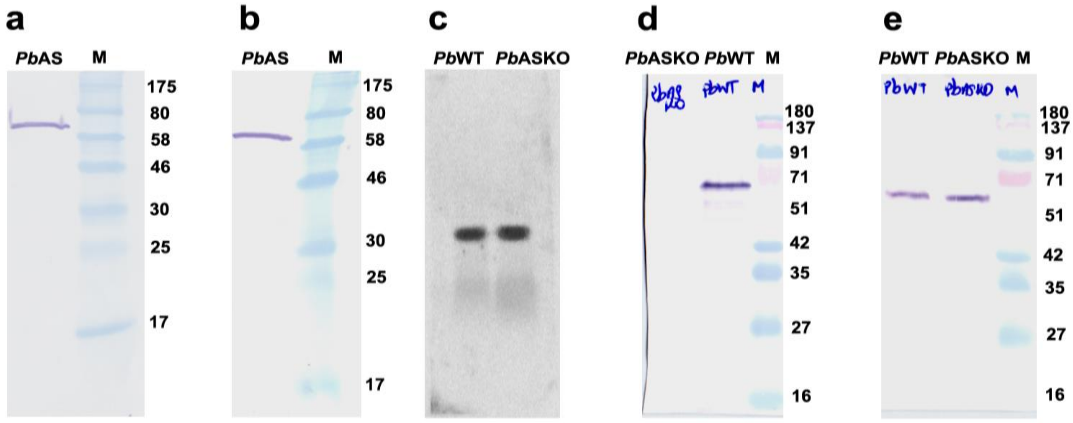

Supplementary Figure 6. Full-length blots representing the images included in Figures 1 and 2. (a) Full-length Western blot for Figure 1d. **(b)** Full-length Western blot for Figure 1e. **(c)** Full-length Northern blot for GAPDH control included in Figure 2e (lower panel). **(d,e)** Full-length Western blots for upper (d) and lower panels (e) given in Figure 2f.
